# An updated overview on the regulatory circuits of polyhydroxyalkanoates synthesis

**DOI:** 10.1111/1751-7915.13915

**Published:** 2021-09-02

**Authors:** Ruchira Mitra, Tong Xu, Guo‐Qiang Chen, Hua Xiang, Jing Han

**Affiliations:** ^1^ State Key Laboratory of Microbial Resources Institute of Microbiology Chinese Academy of Sciences Beijing 100101 China; ^2^ International College University of Chinese Academy of Sciences Beijing 100049 China; ^3^ Center for Synthetic and Systems Biology School of Life Sciences Tsinghua University Beijing China; ^4^ College of Life Science University of Chinese Academy of Sciences Beijing 100049 China

## Abstract

Polyhydroxyalkanoates (PHA) are a promising and sustainable alternative to the petroleum‐based synthetic plastics. Regulation of PHA synthesis is receiving considerable importance as engineering the regulatory factors might help developing strains with improved PHA‐producing abilities. PHA synthesis is dedicatedly regulated by a number of regulatory networks. They tightly control the PHA content, granule size and their distribution in cells. Most PHA‐accumulating microorganisms have multiple regulatory networks that impart a combined effect on PHA metabolism. Among them, several factors ranging from global to specific regulators, have been identified and characterized till now. This review is an attempt to categorically summarize the diverse regulatory circuits that operate in some important PHA‐producing microorganisms. However, in several organisms, the detailed mechanisms involved in the regulation of PHA synthesis is not well‐explored and hence further research is needed. The information presented in this review might help researcher to identify the prevailing research gaps in PHA regulation.

## Graphical abstract

Regulation of PHA synthesis is very diverse, comprising of several regulatory factors. Most PHA‐accumulating microorganisms possess multiple regulatory networks that operate in an integrated manner to regulate PHA synthesis. This review provides an overview on how the PHA synthesis is regulated using some most characterized microbial strains as model organism.

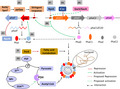

## Introduction

Polyhydroxyalkanoates (PHA) are natural polymers of hydroxyalkanoates produced by microorganisms, including bacteria and archaea, usually under conditions of limited nutritional supply with excess carbon source (Reddy *et al*., 2003; Chen and Wu, 2005). They are accumulated by some microorganism as intracellular carbon and energy reserve to combat adverse environmental conditions (Khanna and Srivastava, 2005). PHA are bioplastics and are gaining considerable prominence in both the environmental and medical fields. Synthesis of PHA is a well‐regulated process that involves a number of enzymes and regulatory proteins (Sagong *et al*., 2018). One of the approaches to maximize PHA production and synthesize novel PHA, is to engineer the biosynthetic pathway (Steinbüchel, 2001). Another approach is to manipulate the regulatory elements controlling PHA synthesis. To realize the latter approach, a better understanding of the regulatory circuits involved in PHA synthesis is necessary. The present review attempts to provide an overview on the regulation of PHA to the readers. It mainly focuses on the current knowledge on PHA regulation in several model species of bacteria and haloarchaea. Understanding the PHA regulation is a progressive topic, and this review might help to identify the prevailing research gaps. Reviews on the regulation of PHA synthesis is limited. The few already published reviews are focussed on the various types of regulations affecting PHA synthesis (Kessler and Witholt, 2001; Velázquez‐Sánchez *et al*., 2020). This review has been presented to the readers from a different point of view. The present review has provided a vivid description of the various regulation systems affecting PHA synthesis mainly with the help of some model organisms.

## An overview on PHA biosynthesis pathways

The type of PHA accumulated is closely related to the microbial species and carbon sources. More than 150 monomeric units of hydroxyalkanoates have been identified as PHA monomers (Steinbüchel and Lütke‐Eversloh, 2003). Depending upon the (carbon atom) chain length in the monomers, PHA are classified as short‐chain length comprising of C3‐C5 monomer (SCL‐PHA), medium‐chain length comprising of C6‐C14 monomer (MCL‐PHA) and long‐chain length consisting of more than 14 carbons in monomer (LCL‐PHA) (Sagong *et al*., 2018). Most researches are focussed on SCL and MCL‐PHA. Synthesis of SCL‐PHA from natural and engineered strains is receiving great attention from researchers due to their wide distribution and high production level (Wang *et al*., 2016). Various Gram negative bacteria genera like *Cupriavidus*, *Burkholderia* and *Azotobacter*, and Gram positive bacteria from genera like *Bacillus*, *Nocardia* and *Rhodococcus*, produce SCL‐PHA. Additionally, a few haloarchaeal species including *Haloferax mediterranei* are SCL‐PHA producers. Three monomers of SCL‐PHA which have received significant consideration are 3‐hydroxybutyrate (3HB), 3‐hydroxyvalerate (3HV) and 4‐hydroxybutyrate (4HB). MCL‐PHA have gained considerable attention as they are more flexible in nature. Most fluorescent *Pseudomonas* species belonging to rRNA homology group I accumulate MCL‐PHA by incorporating 3‐hydroxyalkanoate monomers including 3‐hydroxyhexanoate, 3‐hydroxyoctanoate, 3‐hydroxydecanoate and 3‐hydroxydodecanoate into homo, block‐ or random copolymers (Fiedler *et al*., 2000; Chen and Jiang, 2017).

For synthesis of SCL‐PHA, several PHA monomer supplying pathways using sugars, *n*‐alcohols and fatty acids as carbon sources have been identified in bacteria and haloarchaea (Fig. S1) (Han *et al*., 2013; Jiang *et al*., 2016; Ye *et al*., 2018; Mozejko‐Ciesielska *et al*., 2019). Intermediates derived from these substrates are converted to hydroxyacyl‐CoA, which are polymerized by PHA synthase to form PHA (Sagong *et al*., 2018). Most SCL‐PHA producers accumulate PHB, while some species accumulate copolymers such as poly(3‐hydroxybutyrate‐*co*‐3‐hydroxyvalerate) (PHBV) or poly(3‐hydroxybutyrate‐*co*‐4‐hydroxybutyrate) (P3HB4HB) or poly(3‐hydroxybutyrate‐*co*‐3‐hydroxyvalerate‐*co*‐4‐hydroxybutyrate) (PHBV4HB) (Doi *et al*., 1990; Koller *et al*., 2007). The precursor molecules for 3HB, and 4HB are usually acetyl‐CoA, and succinyl‐CoA, respectively. For 3HV, acetyl‐CoA and propionyl‐CoA are the common precursors. In some cases, intermediates of the β‐oxidation of valerate or other odd‐numbered fatty acids serves as the 3HV precursor (Khanna and Srivastava, 2007). Acetyl‐CoA is a common metabolic intermediate, obtained from glycolytic pathway. Propionyl‐CoA is produced by catabolism of specific amino acids like methionine, threonine by Aspartate/2‐oxobutyrate pathway or by oxidation of odd‐chain fatty acids (Wongkittichote *et al*., 2017). In *H. mediterranei*, four propionyl‐CoA supplying pathways are identified which includes, citramalate/2‐oxobutyrate pathway, the aspartate/2‐oxobutyrate pathway, the methylmalonyl‐CoA pathway and 3‐hydroxypropionate pathway (Han *et al*., 2013). Synthesis of 3HB or 3HV monomers proceed under the action of β‐ketothiolase enzyme, which condenses acetyl‐CoA molecule either with another acetyl‐CoA molecule or propionyl‐CoA molecule to form acetoacetyl‐CoA and/or 3‐ketovaleryl‐CoA (Slater *et al*., 1998). 3‐ketoacyl‐CoA reductase then reduces them to (*R*)‐3‐HB‐CoA and/or (*R*)‐3‐HV‐CoA, respectively. Besides, succinyl‐CoA is an intermediate of the TCA cycle. Succinate semialdehyde dehydrogenase catalyses the conversion of succinyl‐CoA to succinate semialdehyde, which is further oxidized to 4HB by 4‐hydroxybutyrate dehydrogenase (Ye *et al*., 2018). 4HB is further converted to 4HB‐CoA by 4HB‐CoA transferase (Chen *et al*., 2017). Finally, these generated monomers (3HB‐CoA, 3HV‐CoA and 4HB‐CoA) are polymerized to form PHB or its copolymers, PHBV, P3HB4HB and PHBV4HB, catalysed by PHA synthases (Nomura and Taguchi, 2007).

In the case of MCL‐PHA, most monomeric building blocks are *R*‐configured chiral 3‐hydroxyalkanoates. The (*R*)‐3‐hydroxyacyl precursors for the MCL‐PHA monomer in *Pseudomonas* spp. is supplied by two pathways, β‐oxidation pathway and fatty acid *de novo* biosynthesis pathway (Fig. S2) (Mozejko‐Ciesielska *et al*., 2019). In β‐oxidation pathway, acyl‐CoA, generated from fatty acid, is oxidized to enoyl‐CoA, which is converted into (*R*)‐3‐hydroxyacyl‐CoA by enoyl‐CoA hydratase (PhaJ) for PHA synthesis (Tsuge *et al*., 2000). In fatty acid *de novo* biosynthesis pathway, (*R*)‐3‐hydroxyacyl‐CoA precursors are generated from unrelated carbon sources, such as glucose and gluconate. These substrates, by series of reactions, are converted into acetyl‐CoA which is further carboxylated to malonyl‐CoA (Rehm *et al*., 2001). The latter enters the fatty acid *de novo* biosynthesis pathway where it condenses with acyl‐ACP to generate 3‐ketoacyl‐ACP, which is converted to (*R*)‐3‐hydroxyacyl‐ACP by β‐ketoacyl‐ACP reductase (FabG). 3‐hydroxyacyl‐ACP:CoA transacylase (PhaG), actually functions as a 3‐hydroxyacyl‐ACP thioesterase and converts (*R*)‐3‐hydroxyacyl‐ACP to (*R*)‐3‐hydroxyalkanoic acid. Finally, the latter is converted to (*R*)‐3‐hydroxyacyl‐CoA by 3‐hydroxyacyl‐CoA ligase (Wang *et al*., 2012; Hokamura *et al*., 2015). The resulting (*R*)‐3‐hydroxyacyl‐CoA is then polymerized to form MCL‐PHA by PHA synthase.

## Regulation of PHA synthesis

PHA have a high demanding global market owing to their several potential benefits like biodegradability, biocompatibility and thermoplasticity. However, commercial usage of PHA is still limited. One of the primary reasons is the high production cost. Several fermentation strategies have been employed where low cost substrates including agro‐industrial wastes, are utilized to produce PHA at a low cost (Bhattacharyya *et al*., 2015). However, another possibility to reduce PHA production cost is by constructing high yielding PHA producers through genetic manipulation (Zhou *et al*., 2012; Ye *et al*., 2018). PHA metabolism is a bidirectional continuous cycle of synthesis and degradation (Prieto *et al*., 2016). It is an intricate process controlled by complex regulatory circuits where the carbon flow is suitably balanced between the PHA synthesis and the growth of microorganism. Nutrient limitation with excessive carbon supply is an important criterion to promote PHA production. It can be speculated that such kind of growth conditions affect the PHA metabolic pathway and the overall process is controlled by diverse regulatory mechanisms. Among them, PHA synthesis is directly regulated at the enzymatic level. Determination of the kinetic characteristics (*K*_m_, *V*_max_) of the enzymes like β‐ketothiolase, 3‐ketoacyl‐CoA reductase, or PHA synthase enzymes have been studied in PHA producers like *Zoogloea ramigera* (Davis *et al*., 1987; Ploux *et al*., 1988), *Cupriavidus necator* (Steinbüchel and Schlegel, 1991) and *Thermus thermophilus* (Pantazaki *et al*., 2005). These values provide insights on their substrate specificity as well as clues to further improve their activities to promote PHA formation. Allosteric regulation of β‐ketothiolase and PHA synthase has shown to affect PHA accumulation in bacteria like *C. necator* (Oeding and Schlegel, 1973) *Azotobacter beijerinckii* (Senior and Dawes, 1973), *Pseudomonas putida* GPo1 (Ren *et al*., 2009). Likewise, covalent modification of PHA synthase by phosphorylation also affects its enzymatic activity which may further influence PHA accumulation (Juengert *et al*., 2018). Therefore, controlling the enzymatic activity of key proteins involved in PHA biosynthetic pathway is one possible method to regulate PHA metabolism. However, this aspect has not been discussed in detail here as it is not the primary focus of this review.

Another mechanism of controlling PHA metabolism involves the regulatory proteins. PHA granules are covered with proteins which are known as PHA granule‐associated proteins (PGAPs) (Maestro and Sanz, 2017). Some of these PGAPs such as PhaR, PhaM, PhaF and PhaQ often serve as regulatory proteins that bind to their own promoters or the promoters of other PHA biosynthetic genes to regulate their transcription to ensure well‐organized PHA granule formation (Lee *et al*., 2004; Pötter *et al*., 2005; Galán *et al*., 2011; Pfeiffer *et al*., 2011). Thus, modification of these regulatory elements can alter the PHA production level. Additionally, some of the global regulatory systems like Gac/Rsm system, PTS and PTS^Ntr^ system, NtrB/NtrC two‐component regulatory system and quorum sensing also participate in PHA synthesis in some species. Most species have multiple regulatory systems which are intertwined to give a combined effect on PHA synthesis (Table 1). The following sections attempt to discuss the contribution of these regulation models in different PHA‐accumulating bacterial species and haloarchaea.

**Table 1:** Types of regulation present in various microorganisms and their effects on PHA metabolism.

Organism name	Regulator	Description of regulator	Effect on target genes	Effect on PHA metabolism	Reference
*Azotobacter vinelandii*	PhbR	AraC family regulatory protein	PhbR activates the transcription of the *phbBAC* operon.	Inactivation of the *phbR* gene reduced PHB accumulation by 70%.	Peralta‐Gil *et al*. (2002)
	RpoS	Global regulator	RpoS acts as an activator of *phbR* expression. Inactivation of *rpoS* reduced *phbR* and *phbB* transcript level.	Δ*rpoS* showed reduced PHB accumulation.	Hernandez‐Eligio *et al*. (2011)
	CydR	Oxygen‐dependent global regulatory protein	CydR positively impacts the *pha* gene expression.	PHB accumulation in the *cydR* mutant occurred during the exponential phase instead of the stationary phase in wild‐type strain.	Wu *et al*. (2001)
	PTS system	Multicomponent system responsible for uptake and concomitant phosphorylation of carbohydrates	PTS genes affect transcription levels of the *phbR* and *phbBAC* genes possibly by modulating RpoS.	Inactivation of *ptsP* and *ptsO* genes reduced PHB accumulation; inactivation of *ptsN* synthesized PHB twice.	Segura and Espín (1998); Noguez *et al*. (2008); Muriel‐Millán *et al*. (2017)
	GacS/GacA system	Two‐component system	Gac/Rsm system modulates the expression of *phbR* and post‐transcriptionally regulates the expression of *pha* genes.	Deletion of *gacS* gene reduced PHB accumulation.	Castañeda *et al*. (2000); Hernandez‐Eligio *et al*. (2012)
	ArrF sRNA	Azotobacter regulatory RNA involving Fe; iron responsive sRNA	Positive regulator of *phbR* expression during iron limitation; *arrF* inactivation reduced *phbR* and *phbB* transcripts level.	*arrF* inactivation reduced PHB accumulation.	Muriel‐Millán *et al*. (2014)
*Azospirillum brasilense*	NtrB/ NtrC system	Two‐component system	/	Δ*ntrBC* and Δ*ntrC* mutants produced PHB in both exponential phase and stationary phase, irrespective of the nitrogen concentration in the medium.	Sun *et al*. (2000), Sun *et al*. (2002)
*Bacillus megaterium*	PhaQ	DNA‐binding, PHA‐responsive autoregulated repressor	PhaQ negatively regulates *phaP* expression.	PHB possibly acted as an inducer for the PhaQ‐mediated *phaP* regulation.	Lee *et al*. (2004)
*Bradyrhizobium diazoefficiens* USDA110	PhaR	DNA‐binding, PHA‐responsive repressor but not autoregulated	PhaR represses and activates almost 28 genes and 42 genes including *pha* genes; PhaR negatively regulated *phaP1*, *phaP4*, *phaP5*, *phaA1* and *phaA2*, *phaZ1* and phaZ3, *phaC1* and *phaC2* and *fixK2* expression; PhaR activates the expression of *phaB2*; Upregulation of *fixK2* in Δ*phaR* mutant further stimulates the transcription of *phaC2* which results in formation of inactive PHB synthase.	Deletion of PhaR impaired PHB synthesis and increased EPS production. Contrarily, Δ*fixK2* mutant accumulated more PHB compared with wild‐type strain.	Quelas *et al*. (2016); Nishihata *et al*. (2018)
*Burkholderia thailandensis*	Quorum sensing	Global regulator	Loss of QS system reduced *scmR* gene expression which reduced the *phaC* and *phaZ* expression.	Deletion of QS system or *scmR* gene reduced PHA synthesis.	Martinez *et al*. (2020)
*Cupriavidus necator*	PhaR	DNA‐binding, PHA‐responsive autoregulated repressor	PhaR negatively regulates expression of *phaP1* and *phaP3*.	Δ*phaR* mutant led to defective PHA synthesis.	Pötter *et al*. (2002); Pötter *et al*. (2005)
	PhaM	Novel DNA‐binding protein	PhaM acts as a physiological activator of PhaC1.	Deletion of *phaM* gene generated fewer large‐sized PHB granule; distribution of the granules among daughter cells were hampered in Δ*phaM* mutant.	Pfeiffer and Jendrossek (2014); Wahl *et al*. (2012)
	PTS system	Multicomponent system responsible for uptake and concomitant phosphorylation of carbohydrates	/	Inactivation of the *ptsN* gene increased PHB accumulation; inactivation of *ptsI* and *ptsH* led to a reduced PHB accumulation.	Kaddor and Steinbüchel (2011)
*Haloferax mediterranei*	PhaR	DNA‐binding, PHA‐responsive autoregulated repressor	PhaR negatively regulates the *phaRP* operon.	Δ*phaRP* mutant produced significantly reduced amount of PHBV than the Δ*phaP* mutant. Complementation with *phaP* in the Δ*phaRP* mutant partially restored PHBV. Complementation with *phaR* in Δ*phaRP* mutant completely restored PHBV accumulation. However, complementation of both *phaP* and *phaR* resulted into formation of regularly shaped PHBV granules.	Cai *et al*. (2015)
	PPS‐like protein	PPS‐like protein possibly evolved as a regulator protein from the PEP synthetase protein	Deletion of *pps*‐like gene activated the transcription of the three cryptic genes, especially *phaC1*.	Deletion of the *pps*‐like gene resulted to 70.46% increase in PHBV accumulation.	Chen *et al*. (2019); Chen *et al*. (2020)
*Herbaspirillum seropedicae* SmR1	PhaR	DNA‐binding, PHA‐responsive autoregulated repressor	PhaR represses the expression of *phaP1* and *phaP2*. In the wild‐type strain, the two phasins were differentially expressed. The expression level of *phaP2* was 8‐fold lower than *phaP1*. However, *phaP2* expression increased by 6‐fold in the Δ*phaP1* mutant.	PHB synthesis required expression of only PhaP1. However, PhaP2 acts as a backup phasin and ensured PHB synthesis to some extent in Δ*phaP1* mutant.	Kadowaki *et al*. (2011); Alves *et al*. (2016)
	Fnr	Redox‐responsive transcriptional regulator	Deletion of the three Fnr encoding genes reduced the transcription levels of the three *phaC*s (*phaC1*, *phaC2*, and *phaC3*).	Deletion of the three Fnr encoding genes reduced PHB accumulation.	Batista *et al*. (2018)
	NtrB/ NtrC system	Two‐component system	Deletion of *ntrC* upregulated *zwf* gene expression.	Deletion of *ntrC* increased PHB accumulation.	Sacomboio *et al*. (2017)
*Pseudomonas aeruginosa*	RpoN	Global regulator	Deletion of *rpoN* gene inhibited *phaG* gene expression; negative regulator of *phaF* expression.	Δ*rpoN* mutant was unable to accumulate PHA from unrelated carbon sources like gluconate and octanoate, irrespective of nitrogen supply.	Hoffmann and Rehm (2004, 2005)
	Quorum sensing	Global regulator	Deletion of *lasI* or *lasR* or both significantly reduced expression of *phaC1*.	Mutants exhibited reduced PHA content.	Xu *et al*. (2011)
*Pseudomonas chlororaphis* PA23	QS and Anr cross‐regulation	Global regulator	PhzR mediates *anr* gene regulation that modulates *pha* gene expression.	QS‐deficient strain and Δ*anr* mutant accumulated less PHA.	Mohanan *et al*. (2019)
*Pseudomonas extremaustralis*	Anr	Oxygen‐sensitive global regulator	Anr inactivation decreased *phaR* and *phaC* expression by 6,000‐ and 1,380‐fold, respectively.	PHB accumulation under microaerobic and anaerobic conditions reduced significantly.	Tribelli *et al*. (2010)
*Pseudomonas oleovorans*	PhaD	TetR‐like transcriptional regulator	PhaD acts as the transcriptional activator of *phaF*; facilitates *phaI* transcription by binding to its promoter.	Deletion of *phaD* decreased PHA accumulation, reduced granule size and increased granule number.	Klinke *et al*. (2000); Sandoval *et al*. (2007)
*Pseudomonas putida* CA‐3	GacS/GacA system	Two‐component system	Disruption of *gacS* inhibited PhaC1 protein expression.	Δ*gacS* was incapable of PHA accumulation.	Ryan *et al*. (2013)
*Pseudomonas putida* GPo1	PhaF	Intrinsically disordered protein, nucleoid binding ability, PHA granule binding ability, i nvolved in PHA granule segregation	PhaF acts as a negative regulator of *phaC1* gene and *phaIF* operon in PHA non‐accumulating conditions.	Disruption of *phaF* did not affect PHA accumulation under favourable condition.	Prieto *et al*. (1999)
*Pseudomonas putida* KT2440	RpoN	Global regulator	RpoN acts as a negative regulator of *phaF* expression under nitrogen excess conditions.	Δ*rpoN* mutant accumulated more PHA during nitrogen limitation compared with nitrogen excess.	Hoffmann and Rehm (2004, 2005)
	RpoS	Global regulator	RpoS might serve as a negative regulator of * phaC1* promoter.	Deletion of *rpoS* gene increased PHA degradation.	Raiger‐Iustman and Ruiz (2008)
	PsrA	Transcriptional regulator	PsrA is involved in the fatty acid – PHA metabolic network.	Deletion of *psrA* gene reduced the PHA production and also changed the monomer composition.	Fonseca *et al*. (2014)
	Stringent response	Global regulatory system	Deletion of the *relA* and *spoT* genes significantly increased expression of the *phaIF* genes.	PHA production in the Δ*relA/spoT* mutant was similar under both nitrogen limiting and non‐limiting conditions.	Mozejko‐Ciesielska *et al*. (2017)
	PTS system	Multicomponent system responsible for uptake and concomitant phosphorylation of carbohydrates		*ptsP* or *ptsO* deletion impaired PHB accumulation; *ptsN* mutation increased PHB synthesis.	Velázquez *et al*. (2007)
*Pseudomonas putida* KT2442	PhaF	Intrinsically disordered protein, nucleoid binding ability, PHA granule binding ability, involved in PHA granule segregation	Deletion of *phaF* reduced transcription of *phaC1* gene by 3.5‐fold; highest transcriptional level of *phaI* differed from mid‐exponential phase in wild type to stationary phase in the Δ*phaF* mutant.	PhaF acted as an activator of PHA synthesis; PHA content was reduced in the Δ*phaF* mutant during continuous fermentation.	Galán *et al*. (2011)
	PhaD	TetR‐like transcriptional regulator	PhaD acts as a carbon‐ dependent activator of the *pha* gene cluster.	Disruption of *phaD* significantly affected PHA accumulation in the presence of octanoate.	De Eugenio *et al*. (2010)
	Crc	Global regulator	Crc represses *phaC1* expression post‐transcriptionally by inhibiting its translation in nutritionally balanced medium.	Inactivation of *crc* gene increased PHA accumulation in nutritionally balanced medium.	La Rosa *et al*. (2014)
*Pseudomonas putida* KT2442/KT2440	GacS/GacA system	Two‐component system	Disruption of *gacS* reduced transcription rate of the entire *pha* cluster.	Disruption of the GacS sensor kinase was linked to reduced PHA production.	Prieto *et al*. (2016)
*Pseudomonas putida* U	PhaF	Intrinsically disordered protein, nucleoid binding ability, PHA granule binding ability, involved in PHA granule segregation	PhaF might act as a transcriptional activator of *phaC1* gene or enhancer of polymerase activity.	Deletion of *phaF* reduced aliphatic PHA synthesis and completely abolished aromatic PHA synthesis.	Sandoval *et al*. (2007)
*Rhizobium etli*	Ani	Shows 31% and 29% sequence identity with PhaR from *P. denitrificans* and *C. necator*, respectively; possesses putative DNA‐binding domain	Deletion of *aniA* led to disappearance of 795 proteins, including PhaB, resulting into altered global protein expression.	Δ*aniA* showed significant decrease in PHB content and increase in EPS production.	Encarnación *et al*. (2002)
*Sinorhizobium meliloti*	MmgR sRNA	Makes more granules Regulator; noncoding 77‐nucleotide transcript; Belongs to the orthologous sRNAs r8s1 subfamily	MmgR negatively controls phasin gene expression at post transcriptional level.	MmgR finely controlled PHB accumulation. Δ*mmgR* mutant produced abnormally large amounts of PHB and irregularly shaped granules.	Lagares *et al*. (2017)
*Synechocystis* sp. PCC 6803	Slr0058	Novel regulatory protein, structurally similar to PhaF but lacked the DNA binding domain	Slr0058 regulates PHB synthesis initiation and granule formation.	During vegetative growth, no visible PHB granules were detected in Δ*slr0058* mutant; during chlorosis, the mutant had abnormally increased number of PHB granules.	Koch *et al*. (2020b)
	PirC	Novel PII interactor of carbon metabolism	PirC negatively regulates PGAM, encoded by *slr1945* gene	Deletion of *pirC* increased PGAM activity, resulting into increased acetyl‐CoA production and thus over‐accumulation of PHB.	Orthwein *et al*. (2021); Koch *et al*. (2020a)
	SigE	Group 2 RNA polymerase sigma factor	SigE engineering modified the metabolic pathway from glycogen to PHB biosynthesis during nitrogen depletion, overexpression of *sigE* increased the levels of enzymes involved in glycogen catabolism and oxidative pentose phosphate pathway.	Overexpression of *sigE* increased the PHB production levels under nitrogen depletion.	Osanai *et al*. (2013)
	Rre37	OmpR‐type response regulator	Rre37 regulates carbon storage distribution. Overexpression of *rre37* accelerated glycogen catabolism at pathway‐level.	Overexpression of *rre37* enhanced PHB accumulation.	Osanai *et al*. (2014)

“/” Indicates no target genes reported.

### PHA regulation in *Cupriavidus necator*

**Fig. 1:**
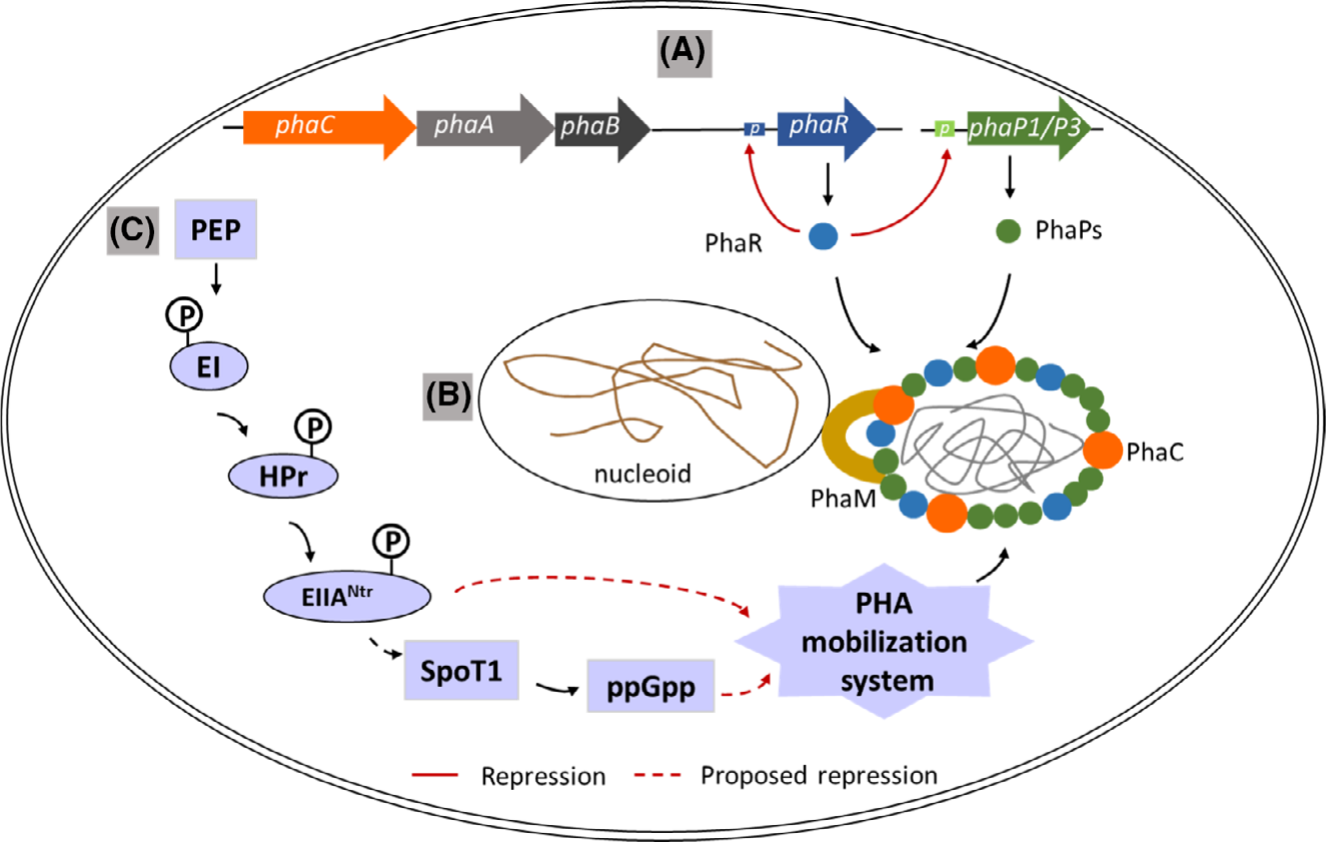
Schematic diagram representing regulation of PHA synthesis in *C. necator*. A. Regulation of PHA synthesis by PhaR. In the absence of PHB chain, free PhaR binds to the promoter of *phaR* and *phaP* and inhibits their transcription. In the presence of nascent PHB chain, PhaR binds to PHB granules and the negative effect of PhaR on *phaR* and *phaP* transcription is released and their transcription continues. As PhaP increases, it displaces PhaR from PHB granules. As free PhaR increases, it again binds to the *phaR* and *phaP* promoter, inhibiting their transcription. B. Association of PHB granule with nucleoid *via* PhaM in *C. necator*. C. Regulation of PHB synthesis by PTS system. PTS system regulates PHB synthesis by modulating PHA mobilization system, possibly by transferring the phosphoryl group or influencing ppGpp level *via* SpoT1. Inactivated PHB degradation may improve PHA accumulation. The red and dashed red arrows indicate repression and proposed repression effects, respectively.

*Cupriavidus necator* is conveniently handled and cultured on cheap feedstock to produce PHB (Raberg *et al*., 2018). The designation of *C. necator* has underwent series of changes. Initially, known as *Hydrogenomonas eutropha*, it has been renamed as *Alcaligenes eutrophus*, *Ralstonia eutropha*, *Wautersia eutropha* and finally as *Cupriavidus necator*. PHB synthesis in this organism is controlled by at least three regulatory factors, PhaR, PhaM and PTS system (Fig. 1).

Phasins are low‐molecular weight amphiphilic proteins, predominantly present on the surface of PHA granules (Steinbüchel *et al*., 1995; Maestro and Sanz, 2017). The hydrophobic domain of PhaP phasin effectively binds to the hydrophobic surface of PHA inclusion body, forming a network‐like layer on PHA granules (McCool and Cannon, 1999). The hydrophilic domain of PhaP remains exposed to the cytoplasm (Maestro and Sanz, 2017). The PhaP expression is regulated by a DNA‐binding protein named PhaR. Co‐occurrence of *phaR* gene and *phaP* gene has been revealed in many bacteria and haloarchaea (Maehara *et al*., 2001; Pötter *et al*., 2002; Segura *et al*., 2003; Chou *et al*., 2009; Kadowaki *et al*., 2011; Cai *et al*., 2012; Nishihata *et al*., 2018). PhaR homologs are well‐distributed among SCL‐PHA‐producing microorganisms and the *phaR* gene is located near the other PHA‐synthesis genes, including *phaC*, *phaA*, *phaB* and *phaZ* in the genome (Maehara *et al*., 2002). The *phaR* gene in *C. necator* is located adjacent to its *phaCAB* gene cluster (Pötter *et al*., 2002). *C. necator* has seven phasin proteins (PhaP1‐PhaP7), among which PhaP1 is the major phasin affecting PHB synthesis and granule size (Pötter *et al*., 2005; Pfeiffer and Jendrossek, 2012). Deletion of only *phaP1* impaired PHB production whereas the other six *phaP* genes had no significant effect (York *et al*., 2001; Pötter *et al*., 2005; Pfeiffer and Jendrossek, 2012). PhaR represses the expression of *phaP1* by binding to its promoter, under PHB non‐accumulating conditions in this strain (Pötter *et al*., 2002; York *et al*., 2002). Additionally, it binds to an intragenic region of *phaP3* and represses its transcription (Pötter *et al*., 2005). Such a regulation of *phaP* genes by PhaR is necessary at the onset of PHB synthesis to avoid the interference by PhaP in the initiation process of PHB synthesis (York *et al*., 2002). Deletion of *phaR* in *C. necator* reduced PHB yield. Intriguingly, the Δ*phaR*Δ*phaP1* double mutant produced further reduced PHB yield compared with Δ*phaP1* mutant, suggesting PhaR also contributes in promoting PHB synthesis in a PhaP‐independent manner in *C. necator* (York *et al*., 2002). Possibly, PhaR represses additional PHA‐synthesis proteins such as PHA synthase whose inaccurate expression might have hindered PHB production or led to premature PHB utilization (York *et al*., 2002; Quelas *et al*., 2016). Once the cells start PHB synthesis, PhaR has the ability to sense the presence of PHB granules and binds to the native PhaP‐free PHB granules, thus releasing the inhibitory effect on *phaP1* and *phaP3* transcription (Pötter *et al*., 2002, 2005). However, the amount of PhaP3 synthesized is lower than PhaP1 which may be due to stronger repression of *phaP3* transcription than *phaP1* by PhaR (Pötter *et al*., 2005). As cells synthesize PhaP1 and small amounts of PhaP3, they gradually cover the PHB granule surface due to their higher affinity towards it and thus PhaR gets gradually detached. During the process of granule formation and polymer chain elongation, the granule surfaces are capable to accommodate both PhaR and PhaPs and thus keeping the cytosolic concentration of free PhaR low enough to allow continuous *phaP1* and *phaP3* transcription. It was observed that Δ*phaP1* mutant synthesized naked PHB granules with their exposed hydrophobic surfaces coalescing to form only one large PHB granule. On the other hand, Δ*phaR* mutant synthesized large amounts of PhaPs that covered the PHB granule and stabilized them, forming smaller sized PHB granules than the wild‐type strain. Thus, it suggests that PhaR‐mediated controlled expression of *phaP* proteins is critical for PHB granule morphogenesis (Pötter *et al*., 2002). Finally, at the end of PHB synthesis process, the PHB granules attain a critical volume and the surface area becomes limited (Quelas *et al*., 2016). Thus, the continuously expressed PhaP1 and PhaP3 proteins displace PhaR, thereby, increasing the cytosolic concentration of free PhaR. These unbound PhaR binds to the *phaP1* and *phaP3* binding sites, again repressing their transcription (Pötter *et al*., 2005). Simultaneously, to inhibit further PhaR expression, the excess unbound PhaR also binds to the promoter region of its own gene and represses its expression. Thus, PhaR is an autoregulated transcriptional repressor which regulates PhaP1, PhaP3 and PhaR expressions and thereby controls PHB biosynthesis in *C. necator* (Fig. 1).

Another PHA granule‐associated protein detected in *C. necator* is PhaM (Fig. 1) (Pfeiffer *et al*., 2011). It is a 32 kDa DNA‐binding protein possessing phasin‐like properties and shows non‐significant similarity (15%) with PhaR protein (Wahl *et al*., 2012). The predicted structure of PhaM reveals a carboxy terminal similar to histone proteins, which explains the DNA binding ability of PhaM (Wahl *et al*., 2012). Its amino terminal contains two potential transmembrane domains which might be involved in the binding with PHB granules. The four lysine residues present in the carboxy terminus PAKKA motifs in PhaM is responsible for attaching PHB granules to the nucleoid region (Bresan and Jendrossek, 2017). Under PHA‐accumulating conditions, PHB granules synthesized by wild type are attached to the nucleoid region (Wahl *et al*., 2012). The association of PHB granules with the nucleoid region is probably related to PhaM. PhaM and PhaC1 are constitutively expressed and PhaM directly interacts with PhaC1 and forms the complex of PhaM‐PhaC1 even in the absence of PHB. The interaction between PhaM and PhaC1 is specific as PhaM is unable to interact with PhaC of *Aeromonas caviae in vitro* although both belong to class I synthases (Ushimaru and Tsuge, 2016). The PhaM‐PhaC1 initiation complex bound to nucleoid serve as a scaffold for PHB granules (Pfeiffer and Jendrossek, 2013, 2014). During cell division and prior to PHB formation, this initiation complex is colocalized at least two discrete nucleoid‐associated foci. As PHB granules formation begin, they attach to the complex following which they distribute among two daughter cells. Thus, each daughter cell receives at least one formed PHB granule. At the later stages of growth, the PhaM and PhaC1 proteins detach from the granules (Bresan and Jendrossek, 2017). The constitutive overexpression of PhaM increased the number of small PHB granules which completely covered the nucleoid surface (Wahl *et al*., 2012). Deletion of *phaM* generated fewer PHB granules with a larger diameter compared with the wild‐type strain. This is probably because PhaM acts as a physiological activator of PhaC1 as one molecule of PhaM activated 10–11 molecules of PhaC1 (Pfeiffer and Jendrossek, 2014). Moreover, the distribution of PHB granules among the daughter cells in the Δ*phaM* mutant was hampered as only one of the daughter cells contained PHB granules (Wahl *et al*., 2012). Thus, PhaM ensures proper segregation of PHB granules into daughter cells during cell division by facilitating the interaction of PHB granules with the nucleoid region. In addition, PhaM also interacts with PhaP5 and forms a PhaM‐PhaP5 complex which are attached to the nucleoid region (Pfeiffer and Jendrossek, 2013). In the wild‐type strain, *phaP5* expression is very low and its deletion did not affect the granule number and localization (Wahl *et al*., 2012). Notably, when *phaP5* was overexpressed, the number of PHB granules increased but their diameter decreased (Wahl *et al*., 2012). Moreover, the PHB granules were detached from the nucleoid region and were localized near both the cell poles in the form of clusters. It is possible that binding of the overexpressed PhaP5 to PhaM, restricts the binding of PhaM to DNA and/or to PhaC1, leading to detachment of PHB granules from nucleoid region. Alternatively, it is also possible that, the overexpressed PhaP5 displaces the PhaM molecules from the PHB granule surface leading to altered subcellular localization of the granules. Therefore, subcellular localization of PHB granules is determined by integrated expressions of PhaM, PhaP5 and PhaC1 in *C. necator*.

Another point of PHA regulation in *C. necator* is exerted by the PTS system (Fig. 1). The sugar PTS system (sugar phosphotransferase system) is a multicomponent system that participates in uptake and concomitant phosphorylation of carbohydrates (Erni, 2013). It’s paralog system is PTS^Ntr^ (nitrogen‐related phosphotransferase system). Phosphoenolpyruvate (PEP) serves as a common phosphoryl donor in both the systems. Sugar PTS system is carbohydrate‐specific where phosphoryl group from PEP is relayed to enzyme I and then to a histidine protein (HPr). Enzyme I and HPr are carbohydrate non‐specific. The phosphoryl group is transferred from HPr to the carbohydrate‐specific enzyme IIA (Krauβe *et al*., 2009). Enzyme IIB accepts the phosphoryl group from IIA and transfers to the sugar attached to another enzyme IIC. PTS^Ntr^ system is nitrogen‐related where the phosphoryl group from PEP is sequentially transferred to nitrogen‐related enzyme I (EI^Ntr^), nitrogen‐related protein (NPr), and then to nitrogen regulatory enzyme II (EIIA^Ntr^) (Wang *et al*., 2005). *C. necator* possesses an incomplete PEP‐PTS system that is composed of enzyme I, HPr and EIIA^Ntr^, EIIA^Man^ encoded by *ptsI*, *ptsH*, *ptsN* and *ptsM*, respectively (Kaddor and Steinbüchel, 2011) (Fig. 1). It lacks EIIB and EIIC components and thus cannot phosphorylate sugars. Intriguingly, the *C. necator* HPr shows 33% amino acid similarity with the NPr of *E. coli* and it strongly phosphorylates enzyme IIA^Ntr^ rather than EIIA^Man^ (Rabus *et al*., 1999; Krauβe *et al*., 2009). Interestingly, IIA^Ntr^ plays a regulatory role in PHB metabolism (Krauβe *et al*., 2009). Inactivation of the *ptsN* gene resulted in a higher PHB accumulation in the mutant strain (Kaddor and Steinbüchel, 2011). Contrarily, deletion of *ptsI* or *ptsH* led to a reduced PHB accumulation when excess carbon source was present. After carbon source was exhausted, the PHB content rapidly reduced in the *ptsI* or *ptsH* mutants. Possibly, the PtsI and PtsH proteins regulate the PHB‐mobilizing system in this strain (Pries *et al*., 1991). In the presence of carbon source, these proteins transfer the phosphoryl group to the PHB mobilizing enzyme system and inactivates the latter. Perhaps, in their absence, the PHB‐mobilizing system cannot be inactivated, leading to  a faster degradation of the accumulated PHB. Alternatively, it is also possible that these proteins regulate PHB mobilization at the transcription level. In 2014, it has been further suggested that the PTS system affects the stringent response in *C. necator* which influences the PHB accumulation (Karstens *et al*., 2014). The stress response during nitrogen limitation is achieved through synthesis of ppGpp nucleotide. SpoT1 and SpoT2 proteins determine the level of ppGpp in cells in response to nitrogen availability. SpoT1 is a bifunctional (p)ppGpp synthase/hydrolase enzyme. However, SpoT2 possesses only ppGpp synthase activity. Δ*spoT1*Δ*spoT2* double mutant showed strongly impaired PHB accumulation and a more active PHB mobilization system compared with the wild‐type and Δ*spoT2* mutant (Juengert *et al*., 2017). On the contrary, simulated Δ*spoT1* mutant constructed by complementing *spoT2* in Δ*spoT1*Δ*spoT2* showed higher PHB accumulation and reduced PHB mobilization. Further evidences suggest that the absence of ppGpp synthesis in Δ*spoT1*Δ*spoT2* triggers PHB mobilization by PhaZa1 PHB depolymerase. On the other hand, a constant high level of ppGpp in the simulated Δ*spoT1* mutant due to the absence of ppGpp hydrolase activity possibly inhibits PhaZa1‐ mediated PHB mobilization. Thus, there exists a connection between stress response and PHB accumulation in *C. necator*. Notably, the non‐phosphorylated EIIA^Ntr^ resulted from *ptsI* or *ptsH* deletion interacts with SpoT1 (Karstens *et al*., 2014). Thus, it is possible that this interaction changes the ppGpp level which affects the PHB content of the mutants.

### PHA regulation in *Pseudomonas putida* KT2440 (or KT2442)

**Fig. 2:**
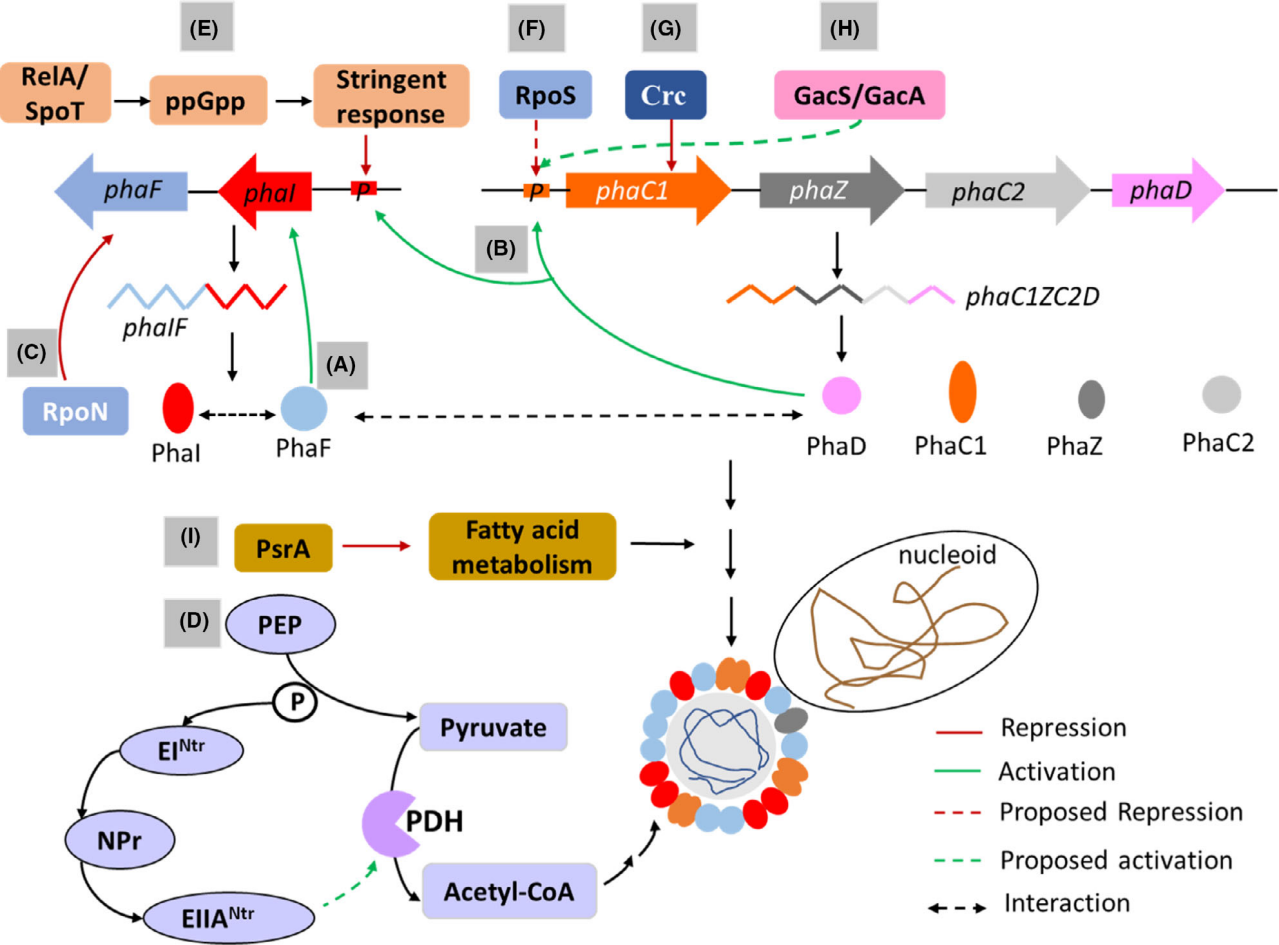
Schematic diagram representing different types of regulation in *P. putida* KT2442/KT2440. A. Positive regulation of *phaC1* and *phaI* expression by PhaF. B. Activation of *phaC1ZC2D* and *phaIF* transcription by PhaD. C. Negative regulation of *phaF* by RpoN under nitrogen excess condition. D. Regulation of PHA synthesis by PTS system. Non‐phosphorylated EIIA^Ntr^ in Δ*ptsP* or Δ*ptsO* inhibits the activity of pyruvate dehydrogenase (PDH) and lowers acetyl‐CoA flux for PHA synthesis. Δ*ptsN* and the Δ*ptsO*Δ*ptsN* mutant enhances PDH activity and increases PHA synthesis by channelling more acetyl‐CoA. E. Negative regulation of *phaIF* operon by stringent response. F. Proposed negative regulation of *phaC1* promoter by RpoS. G. Repression of *phaC1* expression by Crc protein under conditions of nutrient balance. H. Proposed involvement of GacS/GacA system in PHA synthesis. Disruption of the *gacS* sensor kinase reduces transcription rate of the entire *pha* cluster. I. Negative regulation of fatty acid metabolism pathway by PsrA. Deletion of *psrA* genes activates fatty acid metabolism leading to reduced PHA production level. The red and dashed red arrows indicate repression and proposed repression effects, respectively. The green and dashed green arrows indicate activation and proposed activation effects, respectively. The black dashed double arrow represents the existence of interaction.

*P. putida* KT2440 and its spontaneous rifampicin resistant mutant *P. putida* KT2442, are natural MCL‐PHA producers and possesses almost identical expression profile (Follonier *et al*., 2011). In *P. putida* KT2442/KT2440, PhaF, PhaD, Crc, PsrA, RpoN, RpoS, stringent response, PTS system and GacS/GacA system plays regulatory role in PHA synthesis (Fig. 2). The particular effect of the different regulatory factors on PHA synthesis in various other strains of *P. putida* such as *P. putida* GPo1, *P. putida* U and *P. putida* CA‐3 have been summarized in Table 1.

The PHA gene cluster of *P*. *putida* KT2440 comprises of two operons, *phaC1ZC2D* and *phaIF* (Prieto *et al*., 2016). The latter operon is located downstream of the former one. The transcription of *phaC1ZC2D* and *phaIF* are driven by the promoters of *phaC1* and *phaI*, respectively (De Eugenio *et al*., 2010). The *phaC1* and *phaC2* genes encode two type II PHA synthases with the preference for 3‐hydroxyacyl‐CoA with length of C6‐C14, *phaZ* encodes an intracellular PHA depolymerase, *phaD* encodes a TetR‐like transcriptional regulator and *phaI* and *phaF* encode PHA granule‐associated phasin proteins (De Eugenio *et al*., 2010). PhaF is involved in maintaining the molecular architecture of PHA granules (Galán *et al*., 2011; Obeso *et al*., 2015). According to the structural characterization study from Maestro *et al*., (2013), PhaF belongs to the intrinsically disordered protein family. PhaF has an interesting structural property as it possesses three separate motifs (Galán *et al*., 2011; Maestro *et al*., 2013; Tarazona *et al*., 2019). The amino terminal region containing the long and uninterrupted amphipathic alpha‐helical structure is stabilized by the interaction with PHA granules. The carboxy terminus containing histone‐like DNA‐binding domain is natively unfolded in the absence of DNA but acquires a super helical structure upon its non‐specific binding to DNA. It has been demonstrated that PhaF segregates the PHA granules among the two daughter cells during cell division process by binding to both PHA granules and nucleoid region (Galán *et al*., 2011; Tarazona *et al*., 2020). The central core of PhaF contains a leucine zipper motif, which is involved in the oligomerization of PhaF protein. Such kind of coiled‐coil sequence is also predicted in the primary structure of PhaI, which indicated that PhaF and PhaI are likely to form a heterodimer or heterotetramers to stabilize the PHA granules (Maestro *et al*., 2013). *P. putida* KT2442 uses octanoic acid as the preferred carbon source for PHA synthesis since the expression of *phaC1* and *phaI* was higher when octanoic acid was fed than glucose, citric acid or gluconate (Galán *et al*., 2011). PhaF plays a role in regulating the expression of *phaC1* and *phaI*. In the Δ*phaF* mutant, the transcription level of *phaC1* was reduced by 3.5‐fold and the highest transcriptional level of *phaI* was differed from the mid‐exponential phase to the stationary phase compared with the wild type. Moreover, the PHA granules in Δ*phaF* mutant failed to distribute among the daughter cells, which reduced the PHA content during continuous fermentation where PHA formation and cell division take place simultaneously (Galán *et al*., 2011). Hence, PhaF maintains the heterogeneity of the cell population with respect to PHA synthesis and granule distribution. Recently, it has been revealed that PhaF stabilizes PHA granules and functions in properly segregating them during cell division by interacting with the PhaI located on the granule surface (Tarazona *et al*., 2020). Additionally, it is also capable of interacting with PhaD and modulates the regulatory activity of PhaD on transcription of *pha* operon in *P. putida* KT2442. PhaD is not associated with PHA granules rather randomly localized throughout the cytoplasm. PhaD interacts with the *phaI* and *phaC1* promoter regions to activate the transcription of the *phaIF* and *phaC1ZC2D* operon, respectively. (Hoffmann and Rehm, 2004; De Eugenio *et al*., 2010). The upregulation in the transcription levels of the PhaD‐regulated PHA genes is more pronounced when octanoate is used compared with glucose. Thus, PhaD serves as a carbon‐dependent activator of the *pha* gene cluster in *P. putida* KT2442 (De Eugenio *et al*., 2010). It is likely that PhaF may act as a coactivator of PhaD as it has been found that presence of PhaF affected the unilateral binding of PhaD to *phaI* promoter (Fig. 2) (Tarazona *et al*., 2020). However, exact interactive role of PhaF with PhaD and *phaI* is yet to be determined.

There are evidences that *phaIF* and *phaF* transcription in *P. putida* KT2440 are differentially regulated. The *phaF* expression is induced by nitrogen limitation in this strain (Hoffmann and Rehm, 2005). RpoN, RNA polymerase sigma factor 54 (σ^54^), is a global regulator that affects the transcription of nitrogen‐regulated genes. Interestingly in the Δ*rpoN* mutant, *phaF* was expressed irrespective of nitrogen status in the culture. Thus, RpoN acts as a negative regulator of *phaF* expression in *P. putida* KT2440, under nitrogen excess conditions (Fig. 2). In contrast, the co‐transcription of *phaI* and *phaF* was only induced during nitrogen limitation in both the wild‐type and Δ*rpoN* mutant. Thus, the *phaIF* expression is RpoN‐independent. *P*. *putida* KT2440 possesses a typical PTS^Ntr^ system composed of Enzyme I^Ntr^, histidine protein (NPr) and enzyme IIA^Ntr^, encoded by *ptsP*, *ptsO* and *ptsN* genes, respectively (Fig. 2). PHA accumulation was significantly impaired in the case of *ptsP* or *ptsO* deletion (Velázquez *et al*., 2007). However, PHA synthesis increased for *ptsN* deletion. Probably, deletion of *ptsN* gene creates a factual situation of excess carbon source with respect to other limiting nutrients. This channelizes the available carbon source to PHA synthesis. On the contrary, the PHA synthesis machinery of Δ*ptsP* or Δ*ptsO* might sense a shortage of carbon source which directs the carbon source to other metabolic pathways. Intriguingly, the non‐phosphorylated EIIA^Ntr^ inhibits the activity of pyruvate dehydrogenase complex (Pflüger‐Grau *et al*., 2011). But when its encoding gene was deleted, the activity of pyruvate dehydrogenase was improved. It is possible that more acetyl‐CoA is channelled towards PHA synthesis in Δ*ptsN*. Thus, the role of EIIA^Ntr^ in carbon metabolism may exert a metabolic regulation in PHA synthesis in *P. putida* KT2440.

Stringent response is mediated by ppGpp synthetase RelA, and ppGpp synthetase/hydrolase SpoT, encoded by *relA* and *spoT* genes, respectively. Using oleic acid as the carbon source, the wild‐type *P. putida* KT2440 accumulated high amounts of PHA during nitrogen limitation (Mozejko‐Ciesielska *et al*., 2017). However, the production significantly reduced under non‐limiting conditions. Interestingly, the Δ*relA*/*spoT* mutant produced similar levels of PHA in both nitrogen limiting and non‐limiting conditions. Thus, deletion of *relA* and *spoT* genes eliminated the need for nitrogen limitation for PHA synthesis. It was also noted that the expression of *phaIF* genes was significantly higher in Δ*relA*/*spoT*, indicating that the *phaIF* operon was negatively regulated by stringent response.

In the case of nutrient balance, Crc (Catabolite repression control) protein represses *phaC1* expression and inhibits PHA synthesis in *P. putida* KT2442 (La Rosa *et al*., 2014). In nutritionally balanced medium like LB, the *phaC1* expression was strongly inhibited by Crc during the exponential phase. Even addition of excess octanoic acid did not induce *phaC1* expression. However, inactivation of *crc* gene increased the *phaC1* expression by five folds and led to PHA accumulation in small amounts. In the stationary phase, Crc protein is counteracted by two sRNAs, CrcZ and CrcY and its inhibitory effect on *phaC1* was diminished. The strength of Crc‐mediated repression is determined by the level of the two sRNAs, CrcZ and CrcY. Notably, a two‐component system, CbrA‐CbrB, involved in the amino acid uptake and assimilation, influences the transcription of CrcZ and CrcY sRNAs. In the imbalanced medium, CrcZ and CrcY sRNAs completely antagonized Crc protein but in balanced medium, the Crc protein was partially antagonized. Thus, the *phaC1* expression and PHA accumulation was unaffected by Crc protein in imbalanced medium. However, in the balanced medium, deletion of the *crc* gene increased PHA production by 57% during the stationary phase.

The sigma factor RpoS is a global regulator that regulates stress response. RpoS is reported to be involved in PHA metabolism in *P. putida* KT2440. Deletion of *rpoS* gene in this strain increased PHA degradation rate due to higher expression of *phaZ* gene during the stationary phase (Raiger‐Iustman and Ruiz, 2008). Moreover, depletion of the carbon source in this phase lowered the rate of PHA synthesis, resulting in a lower PHA content in the Δ*rpoS*. Hence, it has been suggested that RpoS negatively regulated *phaC1* promoter, possibly in an indirect manner. *P. putida* KT2442/KT2440 also possesses the GacS/GacA system that is involved in the regulation of its PHA synthesis (Prieto *et al*., 2016). Disruption of the GacS sensor kinase led to a reduction in PHA synthesis. The transcription rate of the entire *pha* cluster was reduced. However, the underlying mechanism involved in regulating PHA synthesis by GacS/GacA system is not yet determined. In addition, a transcriptional repressor PsrA negatively regulated the genes involved in fatty acid metabolism pathway in this strain (Fonseca *et al*., 2014). Deletion of *psrA* genes upregulated the *fabB* gene expression and suggested that fatty acid metabolism was more active in Δ*psrA*. Thus, less number of intermediates were channelled towards PHA biosynthesis leading to a reduced production level.

### Complex regulatory circuit in *Azotobacter vinelandii*

**Fig. 3:**
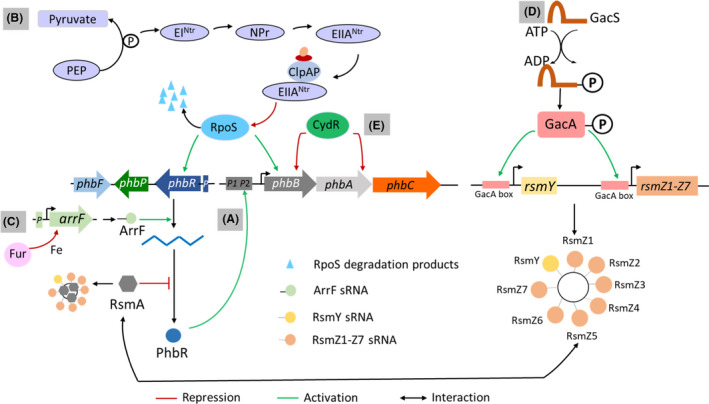
Different regulation models of PHB synthesis in *A. vinelandii*. A. Positive regulation of the transcription of *phbBAC* operon by PhbR. B. Positive regulation of *phbR* and *phbBAC* expression by PTS system and RpoS. Non‐phosphorylated EIIA^Ntr^ induces proteolytic degradation of RpoS by ClpAP complex that lowers the expression level of *phbR*, thereby, diminishing the transcription of the *phbBAC* operon. C. Positive regulation of *phbR* transcription by ArrF sRNA. D. Regulation of PHA synthesis by GacS/GacA system. Phosphorylated GacA activates the transcription of *rsm*Y, and seven *rsm*Z1–*rsm*Z7 genes. These sRNAs bind to the RsmA protein and counteracts its repressive effect on *phbR* translation. E. Negative regulation of PHB synthesis by CydR. The red and green arrows indicate repression and activation effects, respectively. The black double arrow represents the existence of interaction.

*Azotobacter vinelandii* is a free living, nitrogen fixing bacteria capable of accumulating PHB. This species has a complex regulatory circuit which involves PhbR, PEP‐PTS system and Gac/Rsm system (Fig. 3). The genome of *A. vinelandii* possesses a biosynthetic *phbBAC* operon, the regulatory gene *phbR* encoding the transcriptional regulator of *phbBAC* (PhbR), the *phbP* gene and *phbF* encoding the phasin regulator PhbF (known in other bacteria as PhaR) (Segura *et al*., 2000, 2003; Peralta‐Gil *et al*., 2002). Inactivation of the *phbR* gene reduced PHB accumulation by 70%, and the activity of the acetoacetyl‐CoA reductase, β‐ketothiolase and PHB synthase enzymes were reduced by 30%, compared with the wild‐type strain (Peralta‐Gil *et al*., 2002). Thus, PhbR activates the transcription of the *phbBAC* operon. Notably, the transcription of *phbB* gene is driven by two overlapping promoters, pB1 and pB2. In the Δ*phbR* mutant, the level of transcript corresponding to the pB1 promoter was significantly reduced and that corresponding to pB2 promoter increased. This indicates that PhbR activates the transcription of *phbB* driven by pB1 promoter. Possibly, in the absence of PhbR, RNA polymerase more efficiently initiates transcription from pB2 promoter.

Similar to *P. putida* KT2440, *A. vinelandii* possesses PTS^Ntr^ system (Enzyme I^Ntr^, histidine protein NPr and enzyme IIA^Ntr^) which is encoded by *ptsP*, *ptsO* and *ptsN* genes. Inactivation of the *ptsP* or *ptsO* gene reduced PHB accumulation, whereas the *ptsN* inactivation doubled PHB synthesis (Segura and Espín, 1998; Noguez *et al*., 2008). In the case of *ptsP* or *ptsO* inactivation, the terminal phosphoryl acceptor enzyme IIA^Ntr^ is not phosphorylated (Noguez *et al*., 2008). Further inactivation of *ptsN* in these mutants removes the non‐phosphorylated EIIA^Ntr^, which completely or partially restores PHB accumulation. Intriguingly, these *pts* mutations affect the transcription levels of the *phbR* and *phbBAC* genes. The *phbR* mRNA levels and subsequent transcription of *phbBAC* genes in the Δ*ptsP* mutant and Δ*ptsO* mutant are reduced, whereas they increase in the Δ*ptsN* mutant (Noguez *et al*., 2008). It suggests that the non‐phosphorylated EIIA^Ntr^ represses PHB synthesis by downregulating *phbR* and *phbBAC* expression in *A. vinelandii*. It is noteworthy that the *phbR* expression is regulated by the sigma factor RpoS (Hernandez‐Eligio *et al*., 2011). RpoS serves as an activator in the PHB synthesis process as its inactivation decreased the *phbR* and *phbB* transcript levels and subsequently, reduced PHB accumulation (Fig. 3). A deeper insight into the regulatory role of PTS system reveal that non‐phosphorylated EIIA^Ntr^ induces proteolytic degradation of RpoS by ClpAP complex (composed of hexameric ATPase/protein‐unfoldase, ClpA and the tetradecameric proteolytic component, ClpP) in *A. vinelandii* (Fig. 3) (Muriel‐Millán *et al*., 2017). RpoS degradation lowers the expression level of *phbR*, the transcriptional activator of *phbBAC*, thereby, diminishing the transcription of the biosynthetic operon. The *phbR* expression is also post‐ transcriptionally regulated by a small RNA named ArrF (Azotobacter regulatory RNA involving Fe) (Muriel‐Millán *et al*., 2014) (Fig. 3). ArrF links iron concentration in medium and PHA synthesis in *A. vinelandii*. In the condition of iron excess, ferric uptake repressor (Fur) protein, a regulator of iron acquisition, complexes with Fe^2+^ and represses the transcription of *arrF* gene. During iron limitation, this repression is released and *arrF* is transcribed (Jung and Kwon, 2008). Notably, the *phbR* expression is upregulated and PHB synthesis increased during iron‐limiting conditions (Muriel‐Millán *et al*., 2014). The *arrF* inactivation reduced the *phbR* and *phbB* transcripts levels, and PHB accumulation, indicating ArrF sRNA is a positive regulator of *phbR* expression during iron limitation.

GacS/GacA system composed of the sensor kinase (GacS) and the response regulator (GacA) is a two‐component system present in several Gram negative bacteria including *A. vinelandii*. In the presence of appropriate extracellular signal, GacS protein autophosphorylates (Heeb and Haas, 2001). Eventually, GacA is phosphorylated and then acts as a transcriptional activator of several small RNA (sRNA), also known as Rsm (repressor of secondary metabolites) sRNA. These sRNAs further complex with RsmA protein which is a mRNA binding negative translational regulator (Manzo *et al*., 2011). The *rsm* sRNA‐RsmA complex post‐transcriptionally regulates the translation of the target mRNAs and controls protein synthesis. In *A. vinelandii*, Gac/Rsm system regulates the expression of *pha* genes by controlling the post‐transcriptional expression of PhbR (Fig. 3). The phosphorylated GacA activates the transcription of one *rsm*Y, and seven *rsm*Z1–*rsm*Z7 genes (Manzo *et al*., 2011). These sRNAs bind to the RsmA protein and counteracts its repressor effect. Deletion of *gacS* gene resulted in reduced PHB accumulation, indicating that the GacS/GacA cascade plays a positive role in the PHB synthesis (Castañeda *et al*., 2000). While *rsmA* deletion increased the PHB production by 25%, indicating that RsmA play a negative role in PHB synthesis (Hernandez‐Eligio *et al*., 2012). Notably, the transcript levels of *phbR* and *phbB* was reduced in Δ*gacA* mutant, whereas they increased in Δ*rsmA* mutant. This suggests that GacS/GacA system counteracts the RsmA repressor and promotes *phbR* translation which further activates transcription of *phbBAC*. On contrary, deletion of *gacA* reduces the *rsm* sRNA transcription level, which is insufficient to counteract the repressor effect of RsmA protein. Therefore, binding of the free RsmA protein to 5’ leader region of *phbR* transcripts leads to degradation of the latter and hence reduces PhbR protein expression, and subsequent PHB production (Hernandez‐Eligio *et al*., 2012).

Another possible regulatory protein involved in PHA metabolism of *A. vinelandii* is CydR (Fig. 3). It is an oxygen‐dependent global regulatory protein. This FNR‐like transcription factor necessary for the regulation of cytochrome bd expression in *A. vinelandii* (Wu *et al*., 1997). Interestingly, a putative CydR binding site is present upstream of *phbB* in this strain (Setubal *et al*., 2009). The Δ*cydR* mutant showed increased β‐ketothiolase and acetoacetyl‐CoA reductase activity, compared with the wild‐type strain (Wu *et al*., 2001). PHB was accumulated in the Δ*cydR* mutant throughout the exponential phase, whereas wild‐type accumulated PHB only during the stationary phase. Thus, deletion of *cydR* imparts a positive impact on the expression of PHA genes and PHA synthesis in *A. vinelandii*.

### Specific regulator‐mediated regulation in haloarchaea

**Fig. 4:**
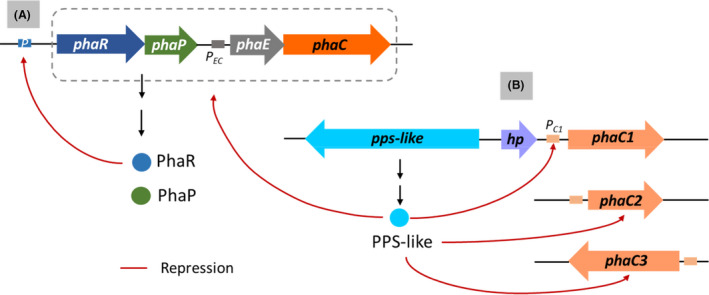
Regulation of PHBV synthesis in *H. mediterranei*. A. PhaR negatively regulates its own and *phaP* expression by binding to their common promoter. B. Negative regulation of *pha* genes by PPS‐like protein. The red arrow indicates repression effect.

The genomes of several haloarchaeal species including *H. mediterranei*, *Haloarcula hispanica*, *Haloarcula marismortui*, *Halomicrobium mukohataei*, *Halorhabdus tiamatea*, *Haloterrigena turkmenica* and *Halopiger xanaduensis*, harbour a conserved *pha* gene cluster, *phaJ1phaRPEC* (Cai *et al*., 2012). Among them, *H. mediterranei* is a natural PHBV producer (Koller *et al*., 2008) and its PHBV synthesis and regulation have been systematically characterized (Lu *et al*., 2008; Han *et al*., 2012; Chen *et al*., 2020). *H. mediterranei* possesses a set of novel enzymes/pathways for PHBV synthesis that are quite distinct from their bacterial counterparts. The precursors for PHBV monomers are acetyl‐CoA and propionyl‐CoA. Acetyl‐CoA is produced *via* glycolysis and propionyl‐CoA is supplied by four different pathways, citramalate/2‐oxobutyrate pathway, the aspartate/2‐oxobutyrate pathway, the methylmalonyl‐CoA pathway and 3‐hydroxypropionate pathway (Han *et al*., 2013). The two precursors are condensed by two novel β‐ketothiolases enzymes (BktB and PhaA). BktB condenses two acetyl‐CoA to acetoacetyl‐CoA, whereas PhaA condenses acetyl‐CoA and propionyl‐CoA to form 3‐ketovaleryl‐CoA (Hou *et al*., 2013). Unlike the tetrameric bacterial β‐ketothiolases that consists of identical subunits, each BktB and PhaA is composed of two different subunits. The larger subunit (BktBα or PhaAα) is the catalytic subunit and determines the substrate specificity. The smaller subunit (BktBβ or PhaAβ) is essentially involved in the enzymatic activity. Moreover, the catalytic residues (Ser‐His‐His) in BktBα or PhaAα is distinct from that of the bacterial β‐ketothiolases (Cys‐His‐Cys). Next, the acetoacetyl‐CoA reductases (PhaB1 and PhaB2) reduce acetoacetyl‐CoA and 3‐ketovaleryl‐CoA to 3‐hydroxybutyryl‐CoA (3HB‐CoA) and 3‐hydroxyvaleryl‐ CoA (3HV‐CoA), respectively (Feng *et al*., 2010). Finally, the 3‐hydroxyacyl‐CoA (3HA‐CoA) are polymerized into PHBV by a novel class III PHA synthase (PhaEC) composed of two subunits, PhaC and PhaE (Lu *et al*., 2008). Additionally, its genome contains three cryptic *phaC* genes, *phaC1*, *phaC2* and *phaC3*. PhaC1 and PhaC3 individually forms an active PHA synthase with PhaE and leads to PHBV synthesis in a PHA synthase gene deletion mutant, *H. hispanica* PHB‐1 (Han *et al*., 2010). During carbon starvation, enoyl‐CoA dehydratase (encoded by *phaJ1*) mediates degradation of the accumulated PHBV by catalysing the dehydration of (*R*)‐3HA‐CoA into enoyl‐CoA which finally enters the β‐oxidation pathway. So far, the regulatory proteins that are known to be involved in PHBV synthesis in *H. mediterranei* are PhaR and PPS (PEP synthetase)‐like proteins (Fig. 4) (Cai *et al*., 2015; Chen *et al*., 2020).

The *phaP* gene located upstream of the *phaEC* encodes the major phasin protein in *H. mediterranei*. Knockout of the *phaP* gene significantly reduced PHBV accumulation and affected the number and size of PHA granules in *H. mediterranei* (Cai *et al*., 2012). However, the *H. mediterranei* PhaP shares no sequence similarity with bacterial phasins and its homologs are only distributed among haloarchaea. Probably, the PhaP protein of *H. mediterranei* represents a novel haloarchaeal type phasin family. The *phaP* gene in this strain is co‐transcribed with the *phaR* gene. The promoter region of *phaRP* operon consists of four tandemly repeated sequences (Cai *et al*., 2015). Most likely, this negative‐*cis* element acts as the binding site for the PhaR protein. The haloarchaeal PhaR homologs constitute of an AbrB‐like domain in the C‐terminus, which is critical for the repressor function of PhaR and supposedly acts as the DNA‐binding motif. The PhaR protein acts as a negative transcriptional regulator of *phaRP* operon by specifically interacting with its promoter. Importantly, in haloarchaea, PhaR regulates the transcription of *phaR* and *phaP* by binding to their common promoter. However, in bacteria, *phaP* and *phaR* possesses independent promoters and PhaR interacts to the respective promoters to regulate their expression (Maehara *et al*., 2002; Pötter *et al*., 2002). Strikingly, PhaR promotes PHBV synthesis in *H. mediterranei* in a PhaP‐independent pathway as *phaR* complementation in Δ*phaRP* mutant completely restored PHBV accumulation (Cai *et al*., 2015). Such kind of phenomenon is similar to *C. necator* (York *et al*., 2002). The Δ*phaRP* cells produced only one or two medium‐sized PHA granules, compared with the multiple moderate‐sized PHA granules in the wild‐type strain (Cai *et al*., 2015). Complementation of only *phaR* in Δ*phaRP* led to the synthesis of one or two large‐sized PHA granules. On the other hand, only *phaP* complementation produced several irregularly shaped small‐ or medium‐sized PHA granules. A similar granule morphology was also observed when *phaP* was overexpressed using a strong promoter in Δ*phaRP*. Thus, it was suggested that PhaR controlled the *phaP* expression that might be necessary for synthesis of PHA granules with regular morphology in *H. mediterranei*.

The PEP/pyruvate interconversion in *H. mediterranei* is mediated by *pyk* and *pps* genes (Chen *et al*., 2019). The *pyk* gene encoding pyruvate kinase catalyses the conversion of PEP to pyruvate whereas the reverse reaction is catalysed by PEP synthetase, encoded by *pps* gene. Deletion of the *pps* gene led to a 35.9% increase in PHBV production most likely by channelling more pyruvate towards PHBV synthesis. Intriguingly, *H. mediterranei* genome possesses a novel protein, PPS‐like, that shows high homology with PPS protein. However, this protein is not involved in the PEP/pyruvate interconversion, instead, deletion of the *pps*‐like gene led to a 70.46% increase in PHBV accumulation. It has been speculated that PPS‐like protein evolved as a regulator protein from the PPS protein (Chen *et al*., 2020). Deletion of *pps*‐like gene promoted the expression of the PHA monomer supplying pathway and upregulated the expression of the *phaEC*, *phaR* and *phaP* genes in *H. mediterranei*. It activates the transcription of the three cryptic genes, especially *phaC1* (Chen *et al*., 2020). Moreover, both PhaC1 and PhaC3 forms functional PHA synthase with PhaE and leads to PHBV synthesis in the Δ*pps*‐likeΔ*phaC* mutant of *H. mediterranei*. Intriguingly, PPS‐like protein effectively interacts with the promoter region of the *phaC1* gene and forms protein‐DNA complex. This indicates that PPS‐like protein acts as a repressor of the *phaC1* expression and hence, its gene deletion leads to an upregulation of the *phaC1* expression. Such type of regulatory function of PPS‐like in PHA synthesis is reported for the first time and thus requires further characterization.

### Regulation in other bacteria

#### PhaQ, a transcriptional regulator in *Bacillus megaterium*

PhaQ is a transcriptional regulatory protein identified in *Bacillus megaterium* (Lee *et al*., 2004). PhaQ differs from the PhaR protein of *C. necator* in amino acid sequence, N‐terminal portion, *cis*‐acting element sequence, as well as size (Maehara *et al*., 2002; Pötter *et al*., 2002). It has high amino acid sequence similarity with hypothetical PhaQs from *Bacillus anthracis* and *Bacillus cereus* (Ivanova *et al*., 2003; Lee *et al*., 2004). Possibly, PhaQs constitute a separate class of transcriptional regulator of PHB synthesis in *Bacillus* species. PhaQ in *B. megaterium* negatively autoregulates its own expression by interacting with its own promoter region and interfering the binding of RNA polymerase with its promoter (Lee *et al*., 2004). The *phaQ* gene is located upstream of the *phaP* gene and they are co‐transcribed (McCool and Cannon, 1999; Lee *et al*., 2004). The genome of this strain consists of another *pha* gene cluster, *phaRBC*, transcribed as a tricistronic operon where *phaR* and *phaC* encode heterodimeric PHB synthase subunits. Generally, PhaP proteins are abundant and the concentration of transcriptional regulatory proteins like PhaR or PhaQ is low in PHA‐producing bacteria (Lee *et al*., 2004). In *B. megaterium*, the intergenic region of *phaQ‐phaP* shows no existence of a promoter. Thus, the differential expression of PhaQ and PhaP is possibly because the cell selectively degrades the *phaQ* transcript in the *phaQP* cotranscript by its post‐transcriptional machinery, leaving the *phaP* transcript intact (Lee *et al*., 2004). The expression level of PhaP protein was low in *B. megaterium* with *phaQ* overexpressed. This indicates that PhaQ negatively regulates the expression of *phaP* in *B. megaterium*. PhaQ interacts with DNA and artificial PHB granules *in vitro*. It is also a PHB‐responsive repressor as it could sense the presence of PHB granules *in vivo*. Perhaps, PHB may act as an inducer for *phaP* expression in the PhaQ‐mediated regulation.

#### Novel regulatory proteins in *Synechocystis* sp.

*Synechocystis* sp. PCC 6803 is a PHA‐accumulating cyanobacterium. During nitrogen depletion, this strain undergoes chlorosis in which the cells first accumulate glycogen and later synthesize PHB by metabolizing the intracellular glycogen pool (Koch *et al*., 2019). Two novel proteins, Slr0058 and PirC, are known to regulate PHB synthesis in this strain (Koch *et al*., 2020b; Orthwein *et al*., 2021). Additonally, response regulator Rre37 and SigE also regulates PHB synthesis in this strain. Slr0058 shows structural similarity with regulatory phasin PhaF from *Pseudomonas* spp. but lacks the DNA binding domain (Koch *et al*., 2020b). The Δ*slr0058* mutant synthesizes PHB but strikingly, no visible PHB granules were detected during the vegetative growth; whereas, it had abnormally increased number and improperly aggregated PHB granules during chlorosis. Possibly, Slr0058 is involved in initiation of PHB granule formation. During vegetative growth, Slr0058 aggregates in foci and then disappears during chlorosis. In the absence of Slr0058, the PHB granules aggregates in an uncontrolled manner. Thus, Slr0058 is a novel regulatory protein involved in the controlled PHB synthesis and granule formation in *Synechocystis* sp. PCC 6803.

The other protein PirC negatively regulates the activity of 2,3‐phosphoglycerate‐independent phosphoglycerate mutase (PGAM) catalysing the conversion of 3‐phosphoglycerate to 2‐phosphoglycerate in lower glycolysis leading to acetyl‐CoA production (Orthwein *et al*., 2021). Under sufficient nitrogen concentration, PirC protein complexes with a signal processor protein, PII. However, during nitrogen limitation, PirC is released from the complex and it inhibits the PGAM activity. Thus, the carbon flux is re‐directed towards glycogen synthesis instead of acetyl‐CoA formation. Interestingly, deletion of *pirC* led to increased glycogen catabolism, leading to acetyl‐CoA production and hence, over‐accumulation of PHB under nitrogen limitation in this strain. Further expression of the *phaA* and *phaB* genes from *C. necator*, under the control of a strong promoter, in the *pirC*‐deleted mutant of *Synechocystis* sp. PCC 6803 significantly improved PHB production (Koch *et al*., 2020a). During nitrogen/phosphorus limitation and in the presence of acetate as carbon source, this Δ*pirC* mutant accumulated up to 81% (wt) PHB compared with only 32%(wt) by wild type under alternating light/dark regime. Thus, it is evident that cultivating regulatory mutants in optimized culture conditions is a promising method to maximize PHA production.

SigE, a group 2 RNA polymerase sigma factor, is reported to regulate PHB biosynthesis in *Synechocystis* sp. PCC 6803 (Osanai *et al*., 2013). Overexpression of *sigE* increased PHB production under nitrogen depletion. Moreover, the enzymes involved in glycogen catabolism and oxidative pentose phosphate pathway were up‐regulated. Thus, engineering of *sigE* modified the metabolic pathway from glycogen to PHB biosynthesis during nitrogen depletion. Another regulator that regulates carbon storage distribution in this strain is Rre37. This OmpR‐type response regulator is induced by nitrogen depletion. Overexpression of *rre37* decreased the glycogen level as it accelerated the catabolism of glycogen and enhanced PHB accumulation (Osanai *et al*., 2014). Overexpression of *sigE* and *rre37* further enhanced PHB accumulation. Rre37 preferentially activated *phaA* and *phaB*, whereas SigE activated *phaC* and *phaE* expressions.

#### PHB synthesis regulation in *Herbaspirillum seropedicae* SmR1

NtrB/NtrC system is a two‐component sensor‐activator regulatory system comprising of a signal transduction protein (P_II_ and/or P_z_), effector protein, sensor (NtrB) and regulatory (NtrC) protein. It is the key regulatory system for nitrogen metabolism in bacteria. During nitrogen limitation, the nitrogen sensing protein, uridylyl transferase/uridylyl‐removing enzyme uridylylates P_II_ or P_z_ protein (Persuhn *et al*., 2000). The uridylylated P_II_ or P_z_ protein enables autophosphorylated NtrB to transfer a phosphoryl group to NtrC. NtrC acts as a transcriptional regulator that controls the expression of nitrogen‐dependent genes. In the presence of excess nitrogen, P_II_ or P_z_ protein is non‐uridylylated that inhibits the kinase activity of NtrB. Moreover, it stimulates the phosphatase activity of NtrB that results in non‐phosphorylation of NtrC, thereby inactivating the regulatory function of NtrC (Hervás *et al*., 2010). *Herbaspirillum seropedicae* SmR1 is a PHA‐accumulating nitrogen‐fixing diazotrophic bacterium belonging to β‐Proteobacteria. This strain possesses the components of the NTR system. Deletion of the *ntrC* gene increased the PHB accumulation, irrespective of the nitrogen status (Sacomboio *et al*., 2017). A deeper insight shows that *ntrC* deletion upregulated the expression of *zwf* gene, which encodes G6PDH (glucose‐6‐phosphate dehydrogenase) In the Δ*ntrC* mutant, the G6PDH activity and NADPH/NADP^+^ ratio increased by 2.8‐fold and 2.1‐fold, respectively. The increased NADPH favours PHB biosynthesis as it is a cofactor for PhaB. Moreover, it also creates a higher pool of reducing power and PHB generation acts as a redox sink. Thus, up‐regulation of *zwf* gene is the key factor leading to higher PHB production in the Δ*ntrC* mutant (Sacomboio *et al*., 2017).

There exists a cyclic dependency between PHB synthesis and redox‐responsive transcriptional regulator Fnr, in *H. seropedicae* SmR1. Transcriptional activation of *phaC* genes by Fnr is required for proper PHB synthesis, whereas optimal PHB production is important for maintaining the Fnr activity (Batista *et al*., 2018). Deletion of the three Fnr encoding genes reduced PHB accumulation and the transcription levels of the three *phaC* genes (*phaC1*, *phaC2* and *phaC3*). During oxygen limitation, the absence of PHB in the Δ*phaC1* mutant disturbed the redox balance of the cell as the mutant was unable to maintain the NAD(P)H/NAD(P)^+^ ratios, resulting in increased sensitivity of the cells to oxidative stress. Notably, the redox balance of cell is Fnr‐dependent because synthesis of cytochrome *c* proteins involved in electron transport chain is regulated by Fnr. Thus, redox balance and PHB biosynthesis is tightly controlled in *H. seropedicae* SmR1.

*Herbaspirillum seropedicae* SmR1 also has a special type of PHB synthesis regulation known as responsive backup circuit (RBC). The genome of this strain consists of three genes encoding putative phasins, *phaP1*, *phaP2* and *phaP3* (Alves *et al*., 2016). PhaP1 is the major phasin followed by PhaP2 in wild type, while PhaP3 is very less abundant and detectable only in the Δ*phaP1* mutant. The expression of both the *phaP1* and *phaP2* genes are regulated by the PhaR regulator (Kadowaki *et al*., 2011). PhaR possesses 83% sequence identity with *C. necator* PhaR and is also capable of binding to DNA and PHB granules. It represses the expression strength of its own promoter and *phaP1* and *phaP2* promoters. In the wild‐type strain, the transcription strength of *phaP2* promoter was 8‐fold lower compared with the *phaP1* promoter (Alves *et al*., 2016). Strikingly, the expression of *phaP2* increased by 6‐fold in the Δ*phaP1* mutant. The expression of *phaP2* is not fully activated in wild type but it serves as a backup phasin gene to guarantee proper phasin expression and allows PHB synthesis to some extent in the Δ*phaP1* mutant. Such a regulation type, known as RBC, increases robustness of the microorganisms under unfavourable conditions.

#### Riboregulation in *Sinorhizobium meliloti*

Small RNAs are known to regulate complex metabolic processes by modulating the expression of multiple target genes in response to various stimuli (Beisel and Storz, 2010). *Sinorhizobium meliloti* produces PHB under unbalanced growth conditions. The fate of the carbon source in such unbalanced conditions is strictly regulated by MmgR sRNA in this strain. MmgR (Makes more granules Regulator) is a negative regulator of PHB accumulation (Lagares *et al*., 2017). During growth, depletion of nitrogen source acts as a stimulus for inducing *mmgR* expression. Once MmgR sRNA is activated, it finely tunes the PHB synthesis. It limits the PHA accumulation to an optimum level, despite of the presence of excess carbon source in the medium. Clearly, PHB accumulation was enhanced and granules were irregularly shaped in the Δ*mmgR* mutant. This was accompanied by overexpression of the PhaP1 and PhaP2 proteins. Thus, MmgR sRNA mediated PHB accumulation in *S. meliloti* involves negative post‐transcriptional regulation of PhaP expression.

#### PHA synthesis regulation exerted by Quorum sensing and other global regulators

Quorum sensing (QS) is a global regulatory mechanism of gene expression in response to extracellular concentration of certain signal molecules called autoinducers (Bassler, 2002). The concentration of the autoinducers increase with bacterial cell density, and hence QS system allows bacteria to monitor their population density by altering various gene expressions (Venturi, 2006). Gram negative bacteria use acylated homoserine lactone (AHL) as autoinducer signal molecules. In *Pseudomonas aeruginosa*, QS system *via* LasI/LasR participates in regulating PHA biosynthesis (Xu *et al*., 2011). LasI, an AHL synthase, catalyses the production of *N*‐3‐oxo‐dodecanoyl homoserine lactone (3‐O‐C_12_‐HSL) as a signal molecule which binds to the transcriptional regulator, LasR (Steindler *et al*., 2009). The 3‐O‐C_12_‐HSL interacts with the N‐terminal domain of the LasR protein and induces structural changes that allows the protein to bind to the DNA sequences of target genes and regulate their transcription (Kiratisin *et al*., 2002). Deletion of *lasI* or *lasR* or both in *P. aeruginosa* reduced the PHA content (Xu *et al*., 2011). Moreover, the mutants showed significantly reduced levels of *phaC1* expression. Possibly, LasR mediated QS system downregulates *phaC1* expression that reduces intracellular PHA biosynthesis in *P. aeruginosa*.

*Pseudomonas chlororaphis* PA23 has three distinct QS systems, PhzI/PhzR, CsaI/CsaR and AurI/AurR, among which PhzI/PhzR is known to affect PHA biosynthesis as its deletion led to a reduced PHA production (Nandi *et al*., 2016; Mohanan *et al*., 2019). This strain also possesses another global regulator ANR which is an oxygen‐sensitive transcriptional factor (Sawers, 1991). ANR acts by binding to the conserved *anr* box present in the promoter of its target genes (Mohanan *et al*., 2019). In *P. chlororaphis* PA23, ANR and PhzI/PhzR are subjected to cross‐regulation (Nandi *et al*., 2016). ANR positively regulates *phzI* and *phzR*, whereas PhzR negatively regulates *anr* transcription. Intriguingly, both the QS‐deficient and Δ*anr* mutant showed similar levels of reduced PHA accumulation. However, the *pha* gene expression profiles in the two mutants were different. The downregulation of the *pha* genes including *phaC1*, *phaC2*, *phaZ*, *phaD*, *phaF* and *phaI* were more pronounced in QS‐deficient strain than Δ*anr* mutant. Notably, the promoter region of the *anr* gene possesses a *phz* box which suggests that it is recognized by the PhzR, activated by AHL. Thus, it is possible that the effect of ANR on PHA synthesis is mediated *via* QS system (Mohanan *et al*., 2019). Moreover, as ANR protein is a global regulator affecting multiple genes, it possibly has an overlapping effect on PHB production. It is revealed that *anr* deletion in *P. seudomonas extremaustralis* decreases the oxidative stress resistance and disturbs the redox balance maintenance under limited oxygen supply (Tribelli *et al*., 2013). Furthermore, recent studies show that ANR‐controlled genes include those related to central carbon metabolism and regeneration of NADPH in *P. putida* (Tribelli *et al*., 2019). Taken together, all these factors probably have an added influence resulting in a reduced PHB accumulation in the Δ*anr* mutant of *P. chlororaphis* PA23 (Mohanan *et al*., 2019).

In *Burkholderia thailandensis*, QS is a complex regulatory circuit. The *scmR* gene is a global regulator for synthesis of various secondary metabolites and also represents a key component of the QS system in this strain. Possibly, ScmR controls gene expression by regulating synthesis of AHL signalling molecule, or by directly binding to target genes, or by indirectly modulating gene expression *via* intermediate regulators (Le Guillouzer *et al*., 2020). *B. thailandensis* possesses three LuxI/LuxR‐type QS systems, BtaI1/BtaR1 (QS‐1), BtaI2/BtaR2 (QS‐2) and BtaI3/BtaR3 (QS‐3). Among them, BtaI1, BtaI2 and BtaI3 are LuxI‐type synthases, and BtaR1, BtaR2 and BtaR3 are the LuxR‐type transcriptional regulators (Le Guillouzer *et al*., 2017). In the Δ*btaR1* and Δ*btaR3* mutants, the transcription level of *scmR* gene was low, indicating that it is activated by QS‐1 and QS‐3 (Martinez *et al*., 2020). Interestingly, an interdependence between the QS‐1 and QS‐3 systems exists in this strain where BtaR1 possibly modulates QS‐3 system and *vice versa* (Le Guillouzer *et al*., 2017). *scmR* deletion reduced *phaC* expression and led to 50% decrease in PHA production (Martinez *et al*., 2020). Complementation of the *scmR* gene restored the wild‐type PHA production. Hence, ScmR positively affects PHA accumulation in *B. thailandensis*. Notably, the Δ*btaI3* or Δ*btaR3* mutant showed decreased PHA accumulation which might be due to the reduced expression of *scmR* gene. Thus, QS‐3 positively controls the PHA synthesis probably by modulating the expression of *scmR* gene. Hence, QS‐dependent regulation of the *scmR* gene contributes to the regulation of PHA synthesis in *B. thailandensis* (Martinez *et al*., 2020).

## Conclusions

Bioplastics are an alternative solution to the increasing plastic pollution caused by over usage of synthetic plastics. PHA are promising bioplastics having potential application in various sectors including biomedical, and packaging industries. The main objectives of PHA research is to facilitate synthesis of PHA with novel characteristics and also with characteristics similar to the plastics so as to offer a greener alternative to the existing products. Moreover, to promote commercial PHA production for widespread applications. To achieve these, strain improvement is a basic requirement. Designing microbial strains having improved PHA‐producing capabilities would reduce production cost of bioplastics. Most studies have showed that engineered strains for PHA will increase PHA biosynthesis and their competitiveness. However, genetic modification of organisms can have unpredictable consequences. Transferring gene from one microorganism to another, gene deletion or rearrangement of gene sequences can result into genetic instability or affect other physiological functions of microorganisms that can create obstacle for industrial applications. A single gene may be regulated by several regulatory factors and may control different physiological traits. Therefore, knowledge of regulatory mechanisms is important and might help to design improved strains with better genetic stability. Regulation is an integral part of PHA synthesis. Identifying the key players of PHA regulation and a deeper characterization of the regulatory pathway is necessary. In this review, different regulatory mechanisms involved in PHA synthesis from typical bacteria and haloarchaea have been discussed. Types of regulation in PHA metabolism is quite diverse, ranging from global regulators to specific regulators. Most organisms possessed multiple regulation models which affected the expression of *pha* gene cluster either positively or negatively. Notably, these regulators controlled PHA metabolism in different organisms at different levels. This review provided a simplified idea on how the regulatory models operated in some specific PHAs‐accumulating bacterial and haloarchaeal species. Such information on PHA regulation is crucial as it would direct the PHA researchers to develop improved PHA‐producing microbial strains with the aid of metabolic engineering.

However, not all regulators involved in PHA synthesis have been identified till now. Hence, continued effort and further investigation is needed to elucidate the unknown regulatory circuits present in PHA metabolism. Moreover, the already characterized regulation models have been hardly applied for PHA production improvement. Thus, it is necessary to implement these regulation models in research work to achieve engineered strains with improved PHA production ability.

## Conflict of interest

The authors declare that they have no conflict of interest.

## Supporting information


**Fig. S1**. Natural pathways for SCL‐PHA synthesis. Key enzymes involved are (1) β‐ketothiolase, (2) 3‐ketoacyl‐CoA reductase, (3) Succinate semialdehyde dehydrogenase, (4) 4‐hydroxybutyrate dehydrogenase, (5) 4HB‐CoA transferase, (6) PHA synthase.
**Fig. S2**. Natural pathways for MCL‐PHA synthesis. Key enzymes involved are (1) Enoyl‐CoA hydratase (PhaJ), (2) β‐ketoacyl‐ACP reductase (FabG), (3) 3‐hydroxyacyl‐ACP:CoA transacylase (PhaG), (4) 3‐hydroxyacyl‐CoA ligase, (5) PHA synthase.
