# Supplementary material for: An updated overview on the regulatory circuits of polyhydroxyalkanoates synthesis
Source: Microb Biotechnol. 2021 Sep 2;15(5):1446–70. doi: 10.1111/1751-7915.13915 (PMC9049629; doi:10.1111/1751-7915.13915)
Supplement: Supplementary file 1 — Fig. S1. Natural pathways for SCL‐PHA synthesis. Key enzymes involved are (1) β‐ketothiolase, (2) 3‐ketoacyl‐CoA reductase, (3) Succinate semialdehyde dehydrogenase, (4) 4‐hydroxybutyrate dehydrogenase, (5) 4HB‐CoA transferase, (6) PHA synthase. Fig. S2. Natural pathways for MCL‐PHA synthesis. Key enzymes involved are (1) Enoyl‐CoA hydratase (PhaJ), (2) β‐ketoacyl‐ACP reductase (FabG), (3) 3‐hydroxyacyl‐ACP:CoA transacylase (PhaG), (4) 3‐hydroxyacyl‐CoA ligase, (5) PHA synthase. [file MBT2-15-1446-s001.docx]

**Supplementary Materials**

**An updated overview on the regulatory circuits of polyhydroxyalkanoates synthesis**

Ruchira Mitra^1,2^, Tong Xu^1^, Guo-Qiang Chen^3^, Hua Xiang^1,4*^, Jing Han^1,4*^

**
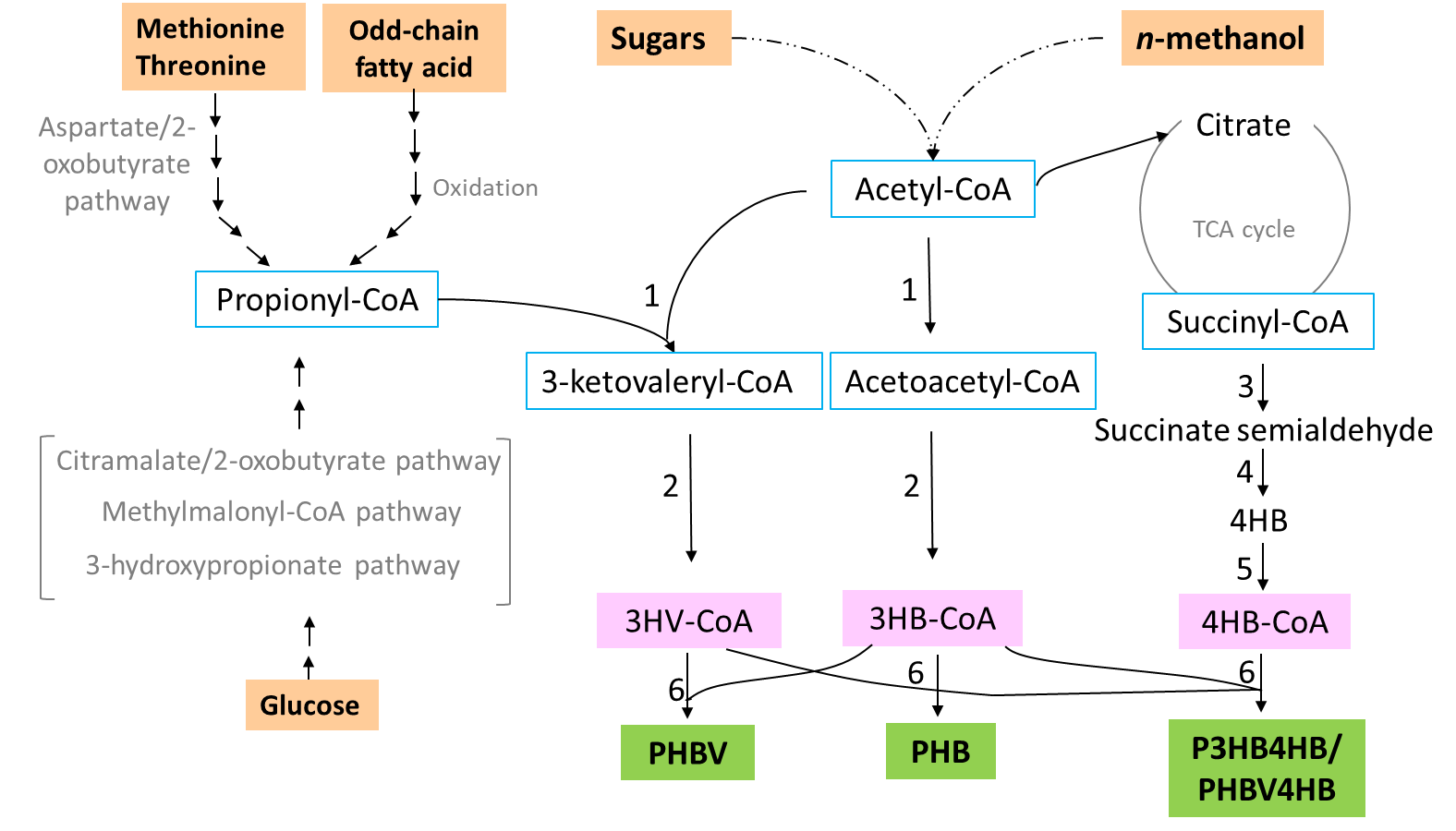
**

**Figure S1.** Natural pathways for SCL-PHA synthesis. Key enzymes involved are (1) β-ketothiolase, (2) 3-ketoacyl-CoA reductase, (3) Succinate semialdehyde dehydrogenase, (4) 4-hydroxybutyrate dehydrogenase, (5) 4HB-CoA transferase, (6) PHA synthase.


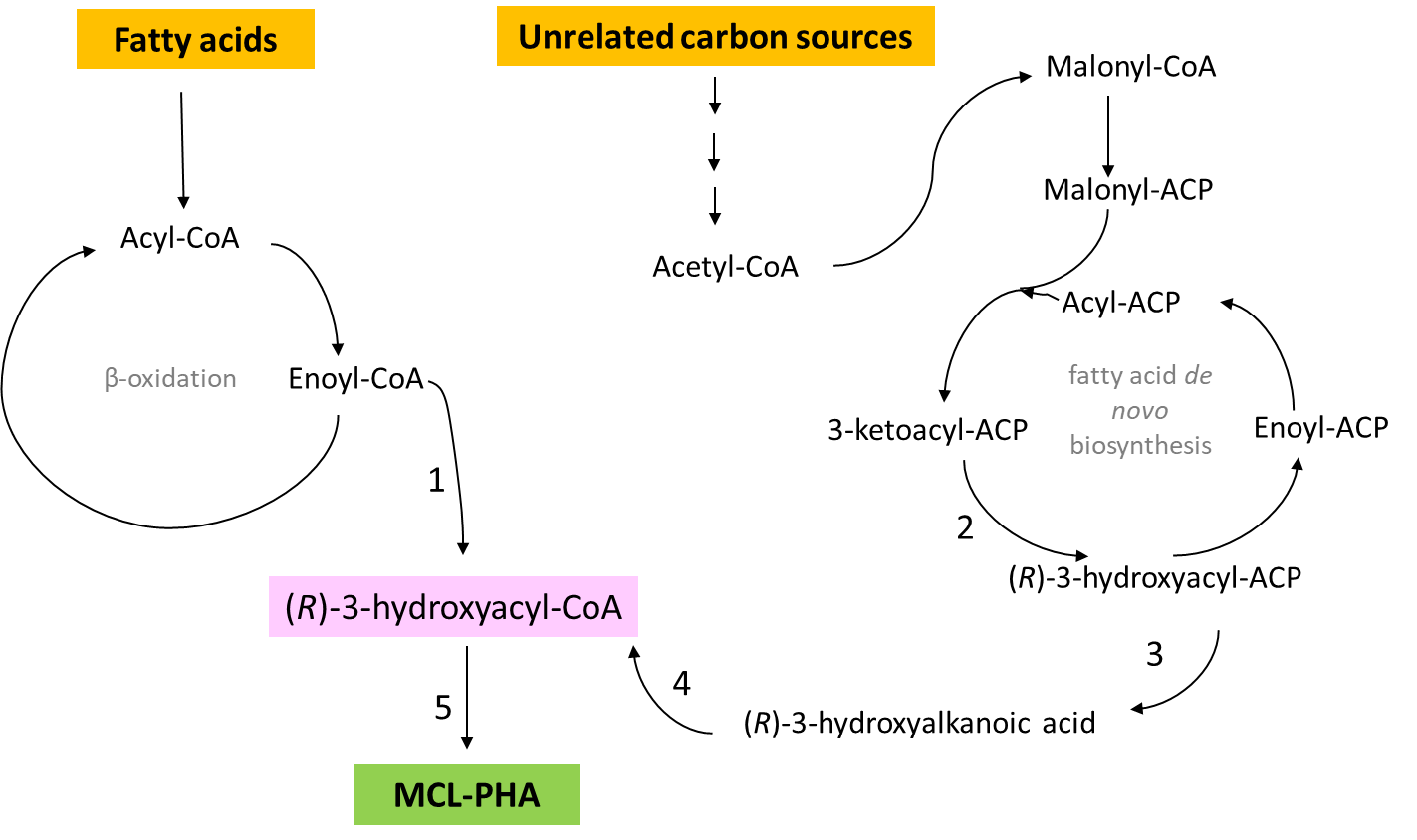


**Figure S2.** Natural pathways for MCL-PHA synthesis. Key enzymes involved are (1) Enoyl-CoA hydratase (PhaJ), (2) β-ketoacyl-ACP reductase (FabG), (3) 3-hydroxyacyl-ACP:CoA transacylase (PhaG), (4) 3-hydroxyacyl-CoA ligase, (5) PHA synthase.
